# RAFTER: a releasing factor tethered RNA editing system for quantifying mRNA translation

**DOI:** 10.26508/lsa.202603651

**Published:** 2026-07-23

**Authors:** Zhiyuan Sun, Peng Dong, Huanyu Yan, Xiaozhen Wen, Yanping Li, Feifei Jiang, Liang Fang, Xi Wang, Wei Chen

**Affiliations:** 1 Shenzhen Key Laboratory of Gene Regulation and Systems Biology, School of Life Sciences, Southern University of Science and Technology , Shenzhen, China; 2 Innovative Center for RNA Therapeutics (ICRT), School of Life Sciences, Southern University of Science and Technology , Shenzhen, China; 3 Department of Systems Biology, School of Life Sciences, Southern University of Science and Technology , Shenzhen, China; 4 Guangming Advanced Research Institute, Southern University of Science and Technology , Shenzhen, China; 5 State Key Laboratory of Reproductive Medicine and Offspring Health, Nanjing Medical University, Nanjing, China; 6 Department of Reproductive Medicine, The First Affiliated Hospital of Nanjing Medical University, Nanjing, China; 7 Department of Urology, The Second Affiliated Hospital of Nanjing Medical University, Nanjing, China

## Abstract

Measurement of mRNA translation level has heavily relied on quantifying the amount of polysome-associated RNAs, which would not always reflect efficient translation. To address this, we developed ReleAsing Factor Tethered Editing of RNA (RAFTER), which combined the RNA editing enzyme with translation releasing factor to selectively label successfully translated RNAs. The translation level estimated based on RAFTER correlated well with that by Ribo-seq and Polysome-seq, but with a greatly simplified workflow, which enables it for single-cell analysis.

## Abstract (precis)

RAFTER combines an RNA editing enzyme with a translation termination factor to selectively label translated RNAs, enabling accurate and scalable translation profiling with single-cell compatibility.

## Introduction

Translational regulation plays a fundamental role in shaping cellular physiology by modulating protein synthesis (1). Dysregulation of mRNA translation has been implicated in numerous diseases, including cancer, neurodegenerative disorders, and developmental abnormalities (2, 3, 4). Therefore, precise and reliable quantification of translation level is crucial for understanding the intricate mechanisms underlying gene expression control. Over the years, several techniques have been developed to measure translation level, each with its own strengths and limitations. Polysome profiling, a classical technique, enables the separation of mRNA molecules bound to varying numbers of ribosomes, with higher numbers indicating more active translation (5). Ribosome footprinting, an alternative tool, involves deep sequencing of ribosome-protected mRNA fragments, which allows determination of the precise positions of ribosomes on individual transcripts (6). Moreover, modified approaches have enabled Ribo-seq analysis in limited-input samples or even at the single-cell level (7, 8, 9), but these methods often require substantial technical optimization, which may limit their broader applicability. To address these challenges, RNA editing–based strategies have recently emerged as powerful tools for measuring ribosome–RNA interactions (10, 11). By coupling deaminase enzymes with small ribosome subunit proteins, these approaches enable in situ labeling of ribosome-associated RNAs and can even be applied to identify ribosome–RNA interactions at single-cell resolution (11, 12 *Preprint*, 13). However, ribosome association does not necessarily indicate productive translation, as ribosome occupancy may also reflect inactive or non-productively engaged ribosomes rather than ongoing protein synthesis (14, 15). To overcome this limitation, we leveraged the translation releasing factor, which integrates into the ribosome complex specifically during the translation termination stage. As translation termination occurs after productive decoding of coding sequences, targeting this stage may potentially reduce signals arising from certain forms of nonproductive ribosome association, thereby providing complementary information on translational regulation.

## Results

**Figure 1.**
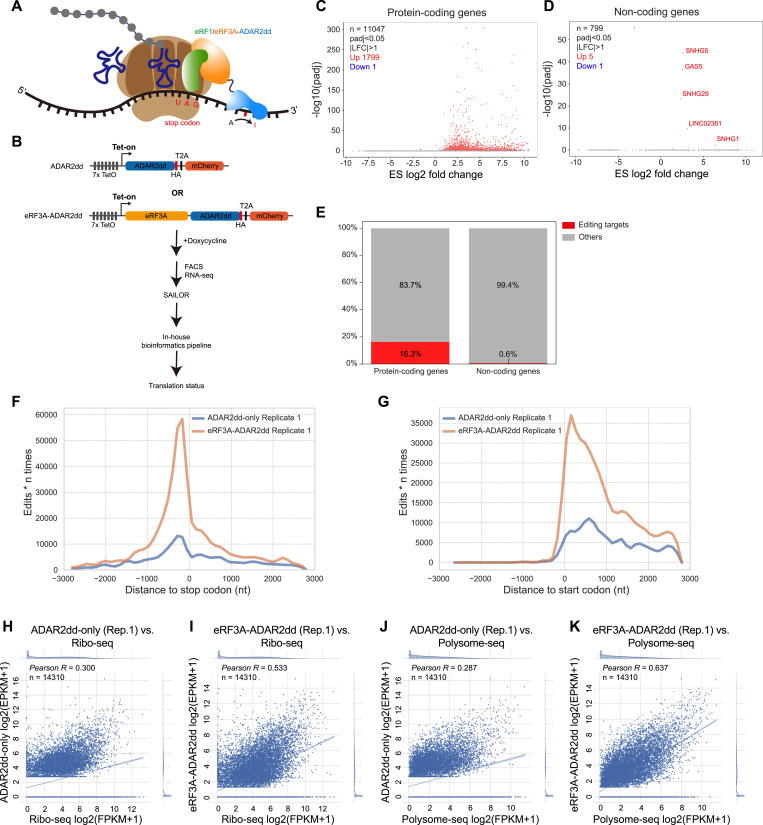
Establishment of RAFTER. **(A)** Schematic representation of the RAFTER method. **(B)** Experimental and analytical workflow of RAFTER. **(C)** Volcano plots comparing the ES of protein-coding genes between cells expressing eRF3a-ADAR2dd and ADAR2dd-only. The X and Y axes represent LFC and −log10(*P*-value), respectively. Statistical significance was calculated using two-sided Fisher’s exact test followed by Benjamini–Hochberg correction. **(D)** Volcano plots comparing the ES of noncoding genes between cells expressing eRF3a-ADAR2dd and ADAR2dd-only. The X and Y axes represent LFC and −log10(*P*-value), respectively. Statistical significance was calculated using two-sided Fisher’s exact test followed by Benjamini–Hochberg correction. **(E)** Bar chart showing the percentages of editing targets identified in protein-coding and noncoding genes, respectively. **(F, G)** Metaplots showing the distribution of confident editing events (≥0.65 confidence score) in the flanking regions of stop codons (F) and start codons (G). **(H, I, J, K)** Scatter plots showing the correlation between RNA editing levels and RNA translation levels. **(H)** ADAR2dd-only (replicate 1) versus Ribo-seq. **(I)** eRF3a-ADAR2dd (replicate 1) versus Ribo-seq. **(J)**, ADAR2dd-only (replicate 1) versus Polysome-seq. **(K)** eRF3a-ADAR2dd (replicate 1) versus Polysome-seq. The number of genes (n) and *Pearson* correlation coefficient (*R*) are indicated in the top left corner of each panel. The diagonal blue lines represent fitted linear regression lines, whereas the surrounding shaded regions indicate the corresponding 95% confidence intervals.

To develop a translation releasing factor–based RNA editing method for quantifying the mRNA translation level, we first analyzed the protein components of the termination complex. The mammalian termination complex is composed of two release factors: eRF1 and eRF3. eRF1 recognizes stop codons and stabilizes the binding of GTP to eRF3 (16), whereas eRF3 is a GTPase that hydrolyzes GTP to stimulate peptide release by eRF1 (17). The cryo-electron microscopy structure of the mammalian termination complex revealed that the N-terminal region of eRF1 reaches deep into the decoding center, whereas eRF3 is located on the surface of the complex (18). Therefore, to mitigate potential structural interference from the release factor–deaminase fusion protein, we selected eRF3 as the candidate. In mammals, there are two distinct *eRF3* genes: the widely expressed *eRF3a* (*GSPT1*) and the tissue-specific *eRF3b* (*GSPT2*) (18). To enable broad applicability, we chose eRF3a and used an eRF3a-ADAR2dd fusion protein, which was expressed in a doxycycline-induced manner (Fig 1A). The method is named ReleAsing Factor Tethered Editing of RNA (RAFTER).

**Figure S1.**
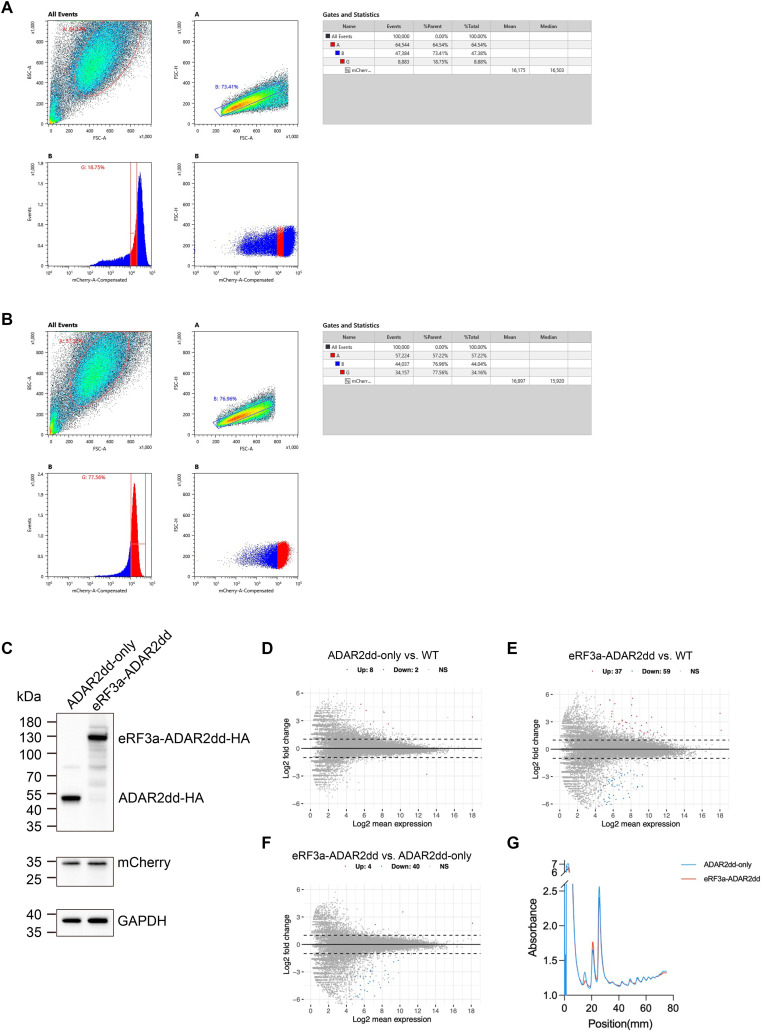
Expression of RAFTER in HEK293T cells. **(A, B)** FACS results of HEK293T cells expressing ADAR2dd-only (A) or eRF3a-ADAR2dd (B). **(C)** Western blot analysis of ADAR2dd-only and eRF3a-ADAR2dd protein levels in FACS-sorted HEK293T cells. The experiment was repeated two times independently with similar results. **(D, E, F)** MA plots showing differential gene expression profiles between the indicated samples. Comparisons are specified at the top of each panel. **(D)** ADAR2dd-only cells versus parental (wild-type, WT) cells. **(E)** eRF3a-ADAR2dd cells versus WT cells. **(F)** eRF3a-ADAR2dd cells versus ADAR2dd-only cells. **(G)** Polysome profiling showing the distribution of RNAs associated with free fractions (non-ribosome bound), 40S ribosomal subunits, 60S ribosomal subunits, 80S monosomes, and polysomes in ADAR2dd-only cells (blue line) and eRF3a-ADAR2dd cells (red line).

In RAFTER, cells expressing ADAR2dd alone were used as controls for background editing. RAFTER expression was induced using the TetON system, and after 48 h of doxycycline treatment, cells exhibiting comparable levels of exogenous gene expression, as determined by mCherry fluorescence intensity through fluorescence-activated cell sorting (FACS), were collected for RNA-seq analysis (Figs 1B and S1A–C). To assess whether expression of the RAFTER system perturbs cellular homeostasis, we compared the transcriptomic profiles of parental, ADAR2dd-expressing, and eRF3a-ADAR2dd–expressing cells. Induction of either construct resulted in only minor transcriptomic changes relative to parental cells (Fig S1D and E). Moreover, direct comparison between eRF3a-ADAR2dd– and ADAR2dd-expressing cells revealed minimal differences in gene expression (Fig S1F). We next examined the impact of RAFTER expression on global translation using polysome profiling. ADAR2dd-expressing and eRF3a-ADAR2dd–expressing cells exhibited nearly identical polysome profiles, indicating that expression of the fusion protein does not measurably alter translational activity (Fig S1G). Collectively, these results demonstrate that eRF3a-ADAR2dd expression, at the level used in this study, does not substantially perturb cellular homeostasis or global translation.

We next used SAILOR (19) to identify confident edit sites and calculated the editing score (ES) for each gene based on edit sites with a confidence score ≥0.65, as previously described (20). Using this strategy, 1,799 annotated protein-coding genes were identified as editing targets, which have significantly higher ES in eRF3a-ADAR2dd–expressing cells than in ADAR2dd-only controls (Fig 1C). In contrast, only five annotated long noncoding RNAs were identified as editing targets (Fig 1D). As expected, the proportion of identified targets was significantly higher among annotated protein-coding genes than among long noncoding RNAs (Fig 1E). Interestingly, four of the five noncoding genes with higher ES in eRF3a-ADAR2dd–expressing cells (*SNHG1*, *SNHG5*, *GAS5*, and *SNHG29*) have previously been reported as translated, supported by Ribo-seq and/or mass spectrometry (21, 22, 23). These together indicate the specificity and sensitivity of our method for detecting translating RNAs.

**Figure S2.**
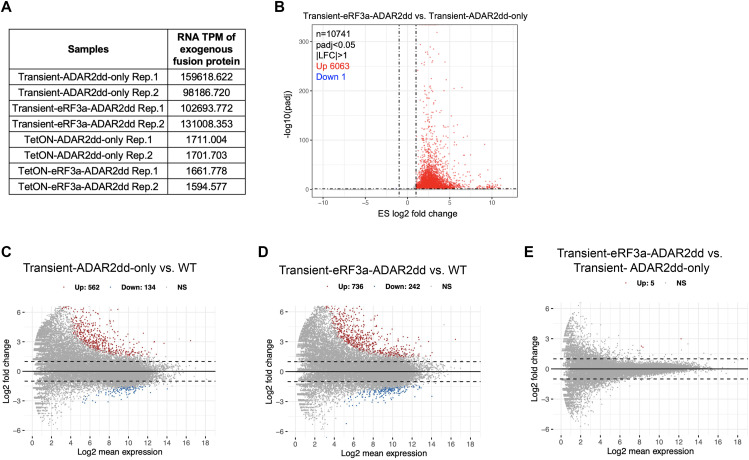
Transient expression of the RAFTER system in HEK293T cells. **(A)** Summary of the RNA TPM of exogenous fusion protein in each sample. **(B)** Volcano plots comparing the ES of noncoding genes between cells transiently transfected with eRF3a-ADAR2dd and ADAR2dd-only plasmids. The X and Y axes represent LFC and -log10(*P*-value), respectively. Statistical significance was calculated using two-sided Fisher’s exact test followed by Benjamini–Hochberg correction. **(C, D, E)** MA plots showing differential gene expression profiles between the indicated samples. Comparisons are specified at the top of each panel. **(C)** Transient-ADAR2dd-only cells versus WT cells. **(D)** Transient-eRF3a-ADAR2dd cells versus WT cells. **(E)** Transient-eRF3a-ADAR2dd cells versus Transient-ADAR2dd-only cells.

Because only 1,799 protein-coding targets were detected under these conditions, we reasoned that the sensitivity of target identification might be limited by editing efficiency, which is influenced by the abundance and activity of the editing enzyme (24). To test this hypothesis, we transiently expressed ADAR2dd or eRF3a-ADAR2dd for 48 h and performed RNA-seq. Transient expression substantially increased deaminase abundance and resulted in a marked increase in target detection, with the number of protein-coding targets rising from 1,799 to 6,063 under the same statistical criteria (Fig S2A and B). These results demonstrate that RAFTER sensitivity is influenced by deaminase expression levels, indicating that the method can capture a significantly broader spectrum of translating RNAs upon optimizing editing efficiency. Notably, although elevated deaminase expression enhanced target detection, it also induced substantial transcriptomic perturbations relative to parental cells (Fig S2C–E). By contrast, the TetON system produced negligible transcriptomic changes while maintaining robust target identification (Fig S1D and E). We therefore used the TetON system for subsequent investigations.

**Figure S3.**
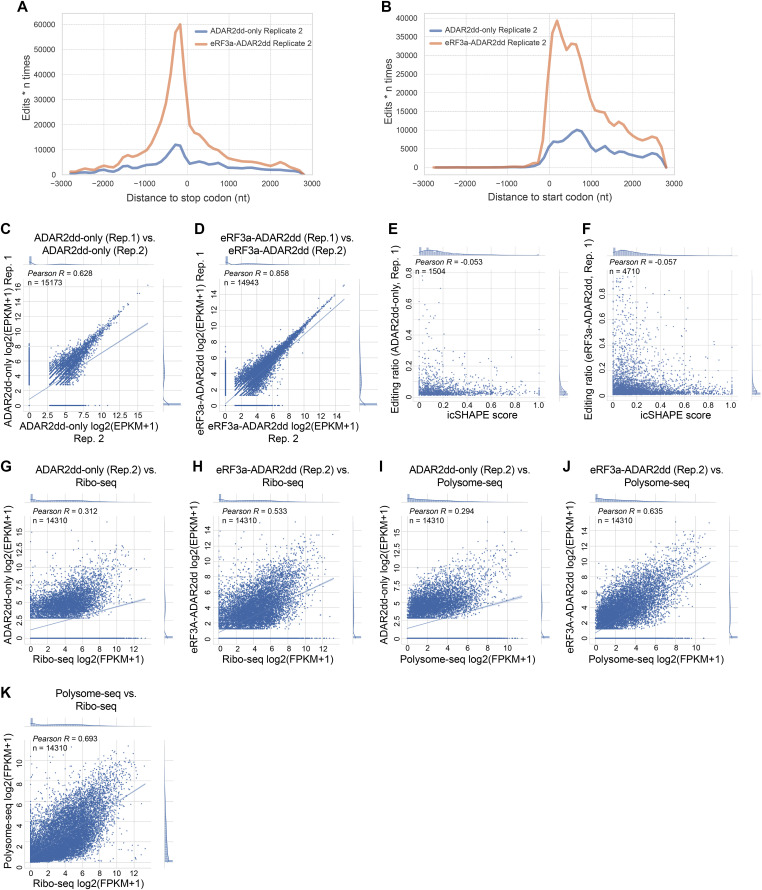
Quantification of RNA editing level. **(A, B)** Metaplots showing the distribution of confident editing events (≥0.65 confidence score) in the second replicate. **(A)** Around the stop codon. **(B)** Around the start codon. **(C, D)** Scatter plots showing the correlation of RNA editing levels between two replicates. **(C)** In ADAR2dd-only cells. **(D)** In eRF3a-ADAR2dd cells. The number of genes (n) and *Pearson* correlation coefficient (*R*) are indicated in the top left corner. The diagonal blue lines represent fitted linear regression lines, whereas the surrounding shaded regions indicate the corresponding 95% confidence intervals. **(E, F)** Scatter plots showing correlations between editing ratios and icSHAPE scores among sites overlapping between editing datasets (first replicate) and published icSHAPE measurements in HEK293T cells. The number of overlapping sites (n) and *Pearson* correlation coefficient (*R*) are indicated at the top right of each panel. **(E)** Editing ratio in ADAR2dd-only cells versus icSHAPE score. **(F)** Editing ratio in eRF3a-ADAR2dd cells versus icSHAPE score. **(G, H, I, J)** Scatter plots showing the correlation between RNA editing levels and RNA translation levels. **(G)** ADAR2dd-only (replicate 2) versus Ribo-seq. **(H)** eRF3a-ADAR2dd (replicate 2) versus Ribo-seq. **(I)** ADAR2dd-only (replicate 2) versus Polysome-seq. **(J)** eRF3a-ADAR2dd (replicate 2) versus Polysome-seq. The number of genes (n) and *Pearson* correlation coefficient (*R*) are indicated in the top left corner. The diagonal blue lines represent fitted linear regression lines, whereas the surrounding shaded regions indicate the corresponding 95% confidence intervals. **(K)** Scatter plots showing the correlation of RNA translation levels measured by Ribo-seq and Polysome-seq. The number of genes (n) and *Pearson* correlation coefficient (*R*) are indicated in the top left corner. The diagonal blue lines represent fitted linear regression lines, whereas the surrounding shaded regions indicate the corresponding 95% confidence intervals.

Because the termination complex binds to mRNAs when the ribosome encounters a stop codon, we next evaluated whether RAFTER-mediated RNA editing could accurately reflect the association between the termination complex and mRNA by plotting the distribution of confident edited reads (see the Materials and Methods section). As expected, eRF3a-ADAR2dd editing resulted in prominent peaks around the stop codon of protein-coding genes, whereas the control protein only generated much sparser editing events (Figs 1F and S3A). Meanwhile, we noticed that eRF3a-ADAR2dd also generated editing peaks around the start codon, with limited extension into the 5′ UTR of mRNAs (Figs 1G and S3B). We hypothesized that this observation may reflect potential spatial proximity between the start and stop codons of mRNAs, representing an additional loop structure distinct from the canonical 5′ cap-3′ poly(A) tail interaction during translation, as previously proposed by Alekhina et al. (25).

Because the majority of editing events generated by eRF3a-ADAR2dd were located within −1,000 to +200 nt relative to the stop codon and −100 to +2,000 nt relative to the start codon, we defined gene-specific windows encompassing these regions to more accurately quantify editing levels (see the Materials and Methods section). The number of editing events within each window was then normalized by both the window length and the total number of editing events across the sample, yielding edits per kilobase per million total edits (EPKM). As shown in Fig S3C and D, although EPKM values correlated well between replicates in both induced ADAR2dd-only and eRF3a-ADAR2dd cells, the *Pearson* correlation coefficient (*R*) was higher in eRF3a-ADAR2dd–expressing cells than in ADAR2dd-only–expressing cells, suggesting that RNA editing events generated by ADAR2dd alone are more stochastic than those generated by eRF3a-ADAR2dd.

It has been reported that local RNA structure can influence ADAR-mediated editing (26). We therefore compared editing ratios of identified confident edit sites with published icSHAPE measurements in HEK293T cells, where lower icSHAPE scores indicate increased RNA structuring (27). Although only a subset of confidently edited sites overlapped with available icSHAPE measurements, no correlation was observed between editing ratio and icSHAPE score in either ADAR2dd-only or eRF3a-ADAR2dd–expressing cells (Fig S3E and F). These findings suggest that local RNA structural accessibility alone cannot account for the editing patterns detected by RAFTER.

Next, we compared EPKM values obtained using RAFTER with FPKM values obtained using ribosome footprinting (Ribo-seq) (28 *Preprint*) and polysome profiling (Polysome-seq) (20), two commonly used measures of translation level. In ADAR2dd-only–expressing cells, EPKM values were poorly correlated (*Pearson R* around 0.3) with FPKM values from either Ribo-seq (Figs 1H and S3G) or Polysome-seq (Figs 1J and S3I). In contrast, in eRF3a-ADAR2dd–expressing cells, these correlations were substantially higher (*Pearson R* around 0.6; Figs 1I and K, and S3H and J), which is comparable to the correlation between Ribo-seq and Polysome-seq (Fig S3K). Together, these results indicate that eRF3a-ADAR2dd–derived EPKM values provide a quantitative measure of translational activity comparable to established translation profiling approaches.

**Figure S4.**
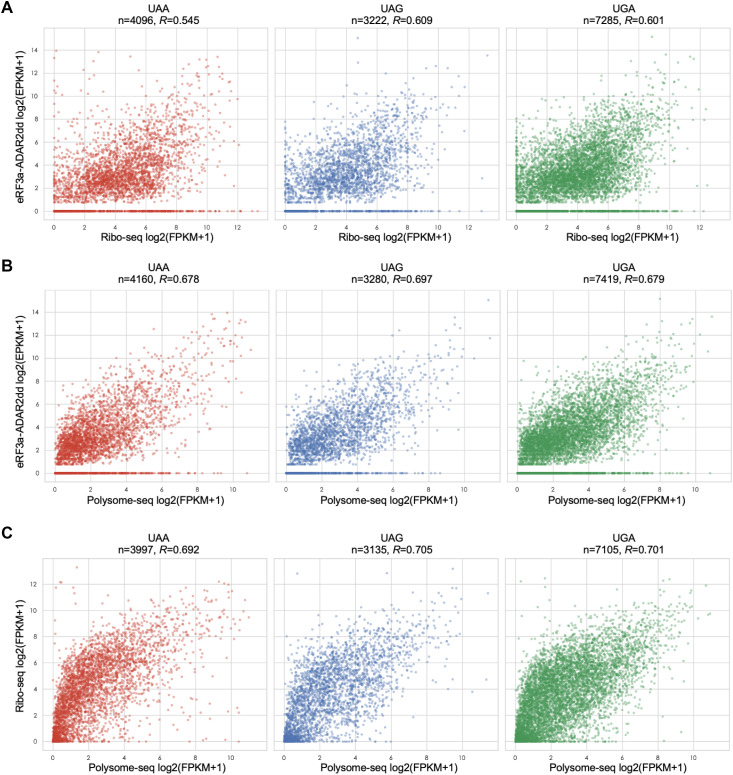
Correlations between RAFTER, Ribo-seq, and Polysome-seq measurements across genes stratified by the identity of different stop codons. **(A, B)** Scatter plots showing correlations between RNA editing levels and translational level among genes grouped by different stop codons. **(A)** eRF3a-ADAR2dd versus Ribo-seq. **(B)** eRF3a-ADAR2dd versus Polysome-seq. **(C)** Scatter plots showing correlations of translational level between Ribo-seq and Polysome-seq among genes grouped by different stop codons. The number of genes (n) and *Pearson* correlation coefficient (*R*) are indicated at the top of each panel.

To further investigate whether stop codon identity (i.e., UAA, UAG, or UGA) influences eRF3a-ADAR2dd signal, we stratified genes by their stop codon types and compared eRF3a-ADAR2dd–derived EPKM values with translation measurements obtained from Ribo-seq and Polysome-seq datasets. eRF3a-ADAR2dd signals showed modestly stronger correlations with both datasets among UAG-terminated transcripts (Fig S4A and B). Interestingly, a similar trend was observed when comparing Ribo-seq and Polysome-seq measurements across the same groups (Fig S4C), suggesting that this pattern is not unique to RAFTER. Overall, these results indicate that stop codon identity has a very subtle effect on the relationship between eRF3a-ADAR2dd signal and translation measurements.

**Figure 2.**
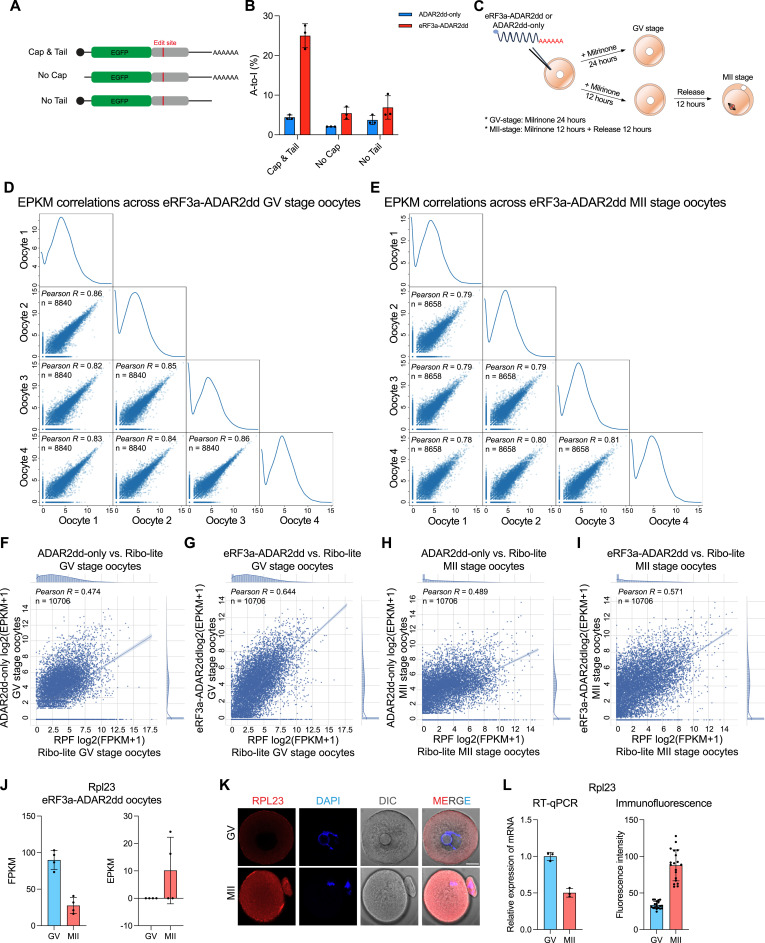
RAFTER captures bona fide translation and translation level in single mouse oocyte. **(A)** Design of different reporter mRNAs. **(B)** Bar plots showing the RNA editing level of different reporter mRNAs. n = 3. *P* (cap and tail) < 0.0001, *P* (no cap) = 0.1491, *P* (no tail) = 0.1890. Statistical significance was calculated using ordinary two-way ANOVA (default in Prism 9). **(C)** Experimental workflow of applying RAFTER on mouse oocytes. **(D)** Scatter plots showing the correlation of EPKM across individual eRF3a-ADAR2dd mRNA-injected GV-stage oocytes. The X and Y axes represent log2(EPKM+1). The number of genes (n) and *Pearson* correlation coefficient (*R*) are indicated in the top left corner of each plot. **(E)** Scatter plots showing the correlation of EPKM across individual eRF3a-ADAR2dd mRNA-injected MII-stage oocytes. The X and Y axes represent log2(EPKM+1). The number of genes (n) and *Pearson* correlation coefficient (*R*) are indicated in the top left corner. **(F, G, H, I)** Scatter plots showing the correlation between RNA editing levels measured by RAFTER and RNA translation levels measured by the Ribo-lite approach in single mouse oocyte. **(F)** In GV-stage oocytes, ADAR2dd-only versus Ribo-lite. **(G)** In GV-stage oocytes, eRF3a-ADAR2dd versus Ribo-lite. **(H)** In MII-stage oocytes, ADAR2dd-only versus Ribo-lite. **(I)** In MII-stage oocytes, eRF3a-ADAR2dd versus Ribo-lite. The X and Y axes represent RPF log2(FPKM+1) and log2(EPKM+1), respectively. The number of genes (n) and *Pearson* correlation coefficient (*R*) are indicated in the top left corner of each panel. The diagonal blue lines represent fitted linear regression lines, whereas the surrounding shaded regions indicate the corresponding 95% confidence intervals. **(J)** RNA level and editing level of *Rpl23* detected in eRF3a-ADAR2dd mRNA-injected oocytes. n = 4. **(K, L)** Independent quantification of RNA and protein level of *Rpl23* in mouse oocytes. **(K)** Representative immunofluorescence results showing protein level of *Rpl23* in mouse oocyte. Scale bar, 20 μm. **(L)** RT-qPCR quantification of RNA level (relative to endogenous 18S rRNA, n = 3) and fluorescence intensity quantification of protein level (from immunofluorescence results, n = 19). *P* (RT-qPCR) = 0.0031. *P* (fluorescence intensity) < 0.0001. Statistical significance was calculated using a two-sided unpaired *t* test (default in Prism 9). The experiment was repeated two times independently with similar results.

**Figure S5.**
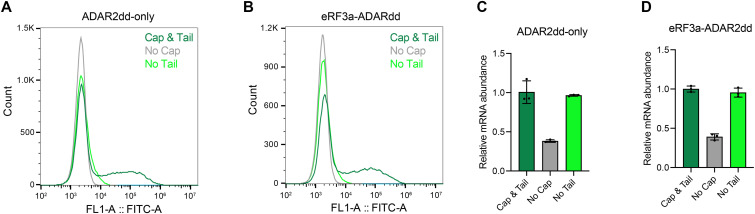
Expression of reporter mRNAs in HEK293T RAFTER cells. **(A, B)** Flow cytometry results of cells expressing reporter mRNAs. **(A)** In ADAR2dd-only–expressing cells. **(B)** In eRF3a-ADAR2dd–expressing cells. **(C, D)** RT-qPCR quantification of RNA levels of reporter mRNAs. RNA levels were normalized by GAPDH. n = 3. **(C)** In ADAR2dd-only–expressing cells. **(D)** In eRF3a-ADAR2dd–expressing cells.

Although RAFTER signals showed strong agreement with established translation profiling approaches, these analyses remained correlative. To directly test whether RAFTER-generated RNA editing reflects bona fide mRNA translation, we designed a reporter system (Fig 2A). Briefly, an endogenous site that was robustly edited in eRF3a-ADAR2dd–expressing cells was cloned into the 3′ UTR downstream of the EGFP coding sequence. EGFP-3′ UTR mRNAs were then synthesized by in vitro transcription with or without a 5′ cap and with or without a 3′ poly(A) tail. The resulting mRNAs were transfected into doxycycline-induced ADAR2dd-only or eRF3a-ADAR2dd–expressing cells for 12 h. Consistent with previous observations (29, 30), flow cytometry analysis showed that only capped and polyadenylated mRNA was efficiently translated (Fig S5A and B). RT-qPCR analysis further indicated that uncapped mRNAs were less stable than capped mRNAs (Fig S5C and D). Through Sanger sequencing, we observed robust A-to-I editing of the capped and polyadenylated mRNA in eRF3a-ADAR2dd–expressing cells, but not in ADAR2dd-only–expressing cells (Fig 2B). Moreover, A-to-I editing levels were substantially lower for mRNAs lacking either the 5′ cap or 3′ poly(A) tail than for the fully modified mRNA in eRF3a-ADAR2dd–expressing cells (Fig 2B). These results together demonstrate that RAFTER reliably reports authentic mRNA translation.

**Figure S6.**
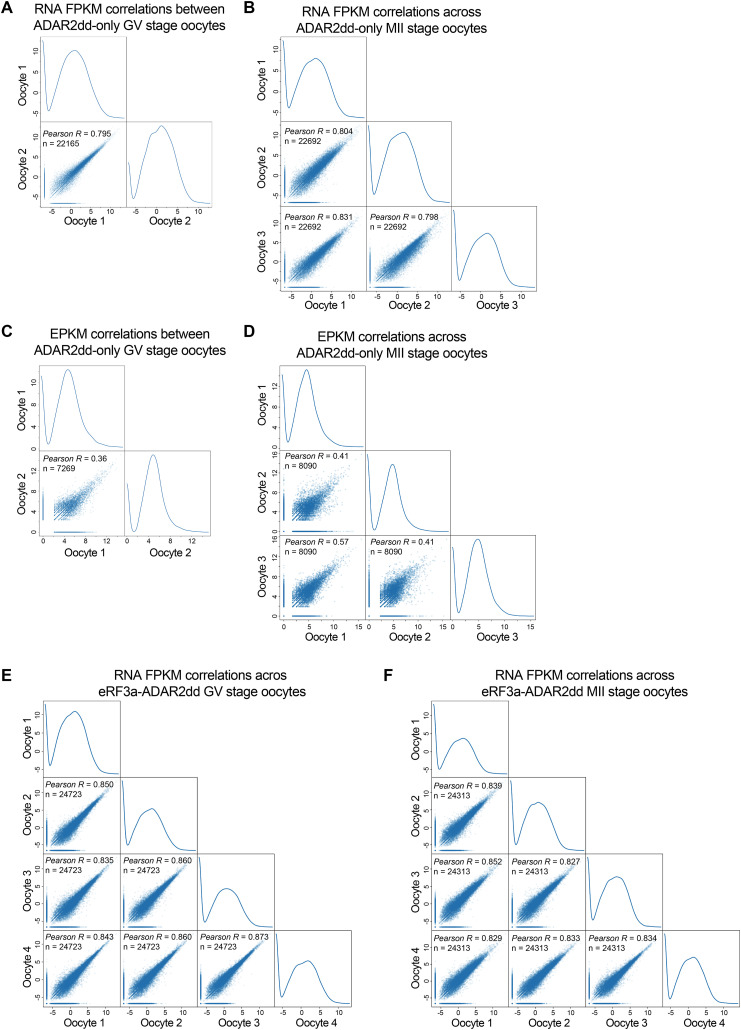
Correlation of RNA expression and editing levels in RAFTER mouse oocytes. **(A, B)** Scatter plots showing the correlation of FPKM across ADAR2dd-only oocytes. The X and Y axes represent log2(FPKM+0.01). The number of genes (n) and *Pearson* correlation coefficient (*R*) are indicated in the top left corner. **(A)** In ADAR2dd-only GV-stage oocytes. **(B)** In ADAR2dd-only MII-stage oocytes. **(C, D)** Scatter plots showing the correlation of EPKM across ADAR2dd-only oocytes. The X and Y axes represent log2(EPKM+1). The number of genes (n) and *Pearson* correlation coefficient (*R*) are indicated in the top left corner. **(C)** In ADAR2dd-only GV-stage oocytes. **(D)** In ADAR2dd-only MII-stage oocytes. **(E, F)** Scatter plots showing the correlation of FPKM across eRF3a-ADAR2dd oocytes. The X and Y axes represent log2(FPKM+0.01). The number of genes (n) and *Pearson* correlation coefficient (*R*) are indicated in the top left corner. **(E)** In eRF3a-ADAR2dd GV-stage oocytes. **(F)** In eRF3a-ADAR2dd MII-stage oocytes.

**Figure S7.**
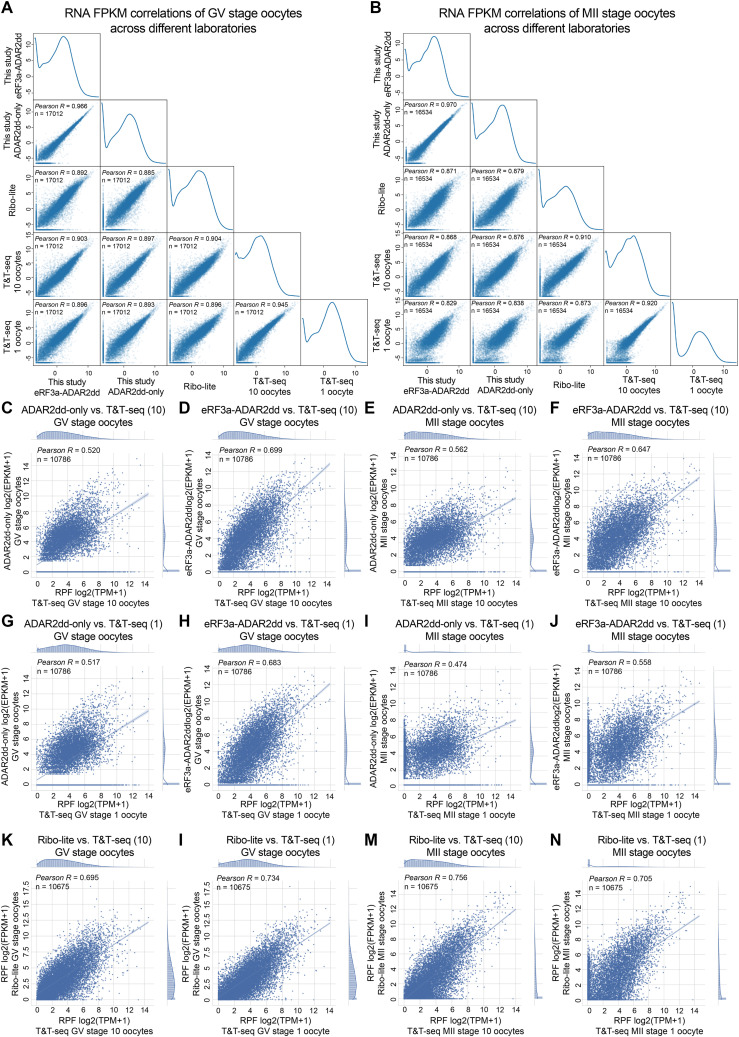
Correlation of RNA expression, editing, and translation levels across mouse oocytes from different laboratories. **(A, B)** Scatter plots showing the correlation of RNA expression levels across oocytes from different laboratories. RNA expression levels from this study and the Ribo-lite approach are represented as log2(FPKM+0.01). RNA expression levels from the T&T-seq approach are represented as log2(TPM+0.01). The number of genes (n) and *Pearson* correlation coefficient (*R*) are indicated in the top left corner. **(A)** GV-stage oocytes. **(B)** MII-stage oocytes. **(C, D, E, F)** Scatter plots showing the correlation between RNA editing levels measured by RAFTER in a single mouse oocyte and RNA translation levels measured by the T&T-seq approach in 10 mouse oocytes. The X and Y axes represent RPF log2(TPM+1) and log2(EPKM+1), respectively. The number of genes (n) and *Pearson* correlation coefficient (*R*) are indicated in the top left corner. The diagonal blue lines represent fitted linear regression lines, whereas the surrounding shaded regions indicate the corresponding 95% confidence intervals. **(C)** In GV-stage oocytes, ADAR2dd-only versus T&T-seq. **(D)** In GV-stage oocytes, eRF3a-ADAR2dd versus T&T-seq. **(E)** In MII-stage oocytes, ADAR2dd-only versus T&T-seq. **(F)** In MII-stage oocytes, eRF3a-ADAR2dd versus T&T-seq. **(G, H, I, J)** Scatter plots showing the correlation between RNA editing levels measured by RAFTER and RNA translation levels measured by the T&T-seq approach in a single mouse oocyte. The X and Y axes represent RPF log2(TPM+1) and log2(EPKM+1), respectively. The number of genes (n) and *Pearson* correlation coefficient (*R*) are indicated in the top left corner. The diagonal blue lines represent fitted linear regression lines, whereas the surrounding shaded regions indicate the corresponding 95% confidence intervals. **(G)** In GV-stage oocytes, ADAR2dd-only versus T&T-seq. **(H)** In GV-stage oocytes, eRF3a-ADAR2dd versus T&T-seq. **(I)** In MII-stage oocytes, ADAR2dd-only versus T&T-seq. **(J)** In MII-stage oocytes, eRF3a-ADAR2dd versus T&T-seq. **(K, L, M, N)** Scatter plots showing the correlation of RNA translation levels measured by the Ribo-lite and T&T-seq approach mouse oocytes. The X and Y axes represent T&T-seq RPF log2(TPM+1) and Ribo-lite RPF log2(FPKM+1), respectively. The number of genes (n) and *Pearson* correlation coefficient (*R*) are indicated in the top left corner. The diagonal blue lines represent fitted linear regression lines, whereas the surrounding shaded regions indicate the corresponding 95% confidence intervals. **(K)** In GV-stage oocytes, Ribo-lite single oocyte versus T&T-seq 10 oocytes. **(L)** In GV-stage oocytes, Ribo-lite single oocyte versus T&T-seq single oocyte. **(M)** In MII-stage oocytes, Ribo-lite single oocyte versus T&T-seq 10 oocytes. **(N)** In MII-stage oocytes, Ribo-lite single oocyte versus T&T-seq single oocyte.

Although single-cell ribosome profiling and mass spectrometry have been developed (7, 8, 9, 31), the technical complexity and high instrumentation demand limit their widespread use. In principle, RAFTER offers a feasible alternative for single-cell detection of mRNA translation. To explore this possibility, we applied RAFTER to measure the translation level of mouse oocytes during the germinal vesicle (GV)-to-metaphase II (MII) transition. In brief, in vitro transcribed eRF3a-ADAR2dd mRNAs or ADAR2dd-only mRNAs were microinjected into oocytes at the GV stage. Injected oocytes were maintained at the GV stage for 24 h in the presence of milrinone (milrinone 24 h) to allow sufficient translation of the injected mRNAs (Fig 2C). For the MII stage, oocytes were released from the milrinone block after 12 h and collected 12 h later (milrinone 12 h + release 12 h) (Fig 2C). We collected four biological replicates for eRF3a-ADAR2dd mRNA-injected oocytes and two to three biological replicates for ADAR2dd-only mRNA-injected oocytes at each stage, which showed high correlation in RNA expression levels between replicates (Fig S6A, B, E, and F). Furthermore, the RNA expression profiles of our samples correlated well with published datasets, supporting the high quality of our single-oocyte RNA-seq libraries (Fig S7A and B).

**Figure S8.**
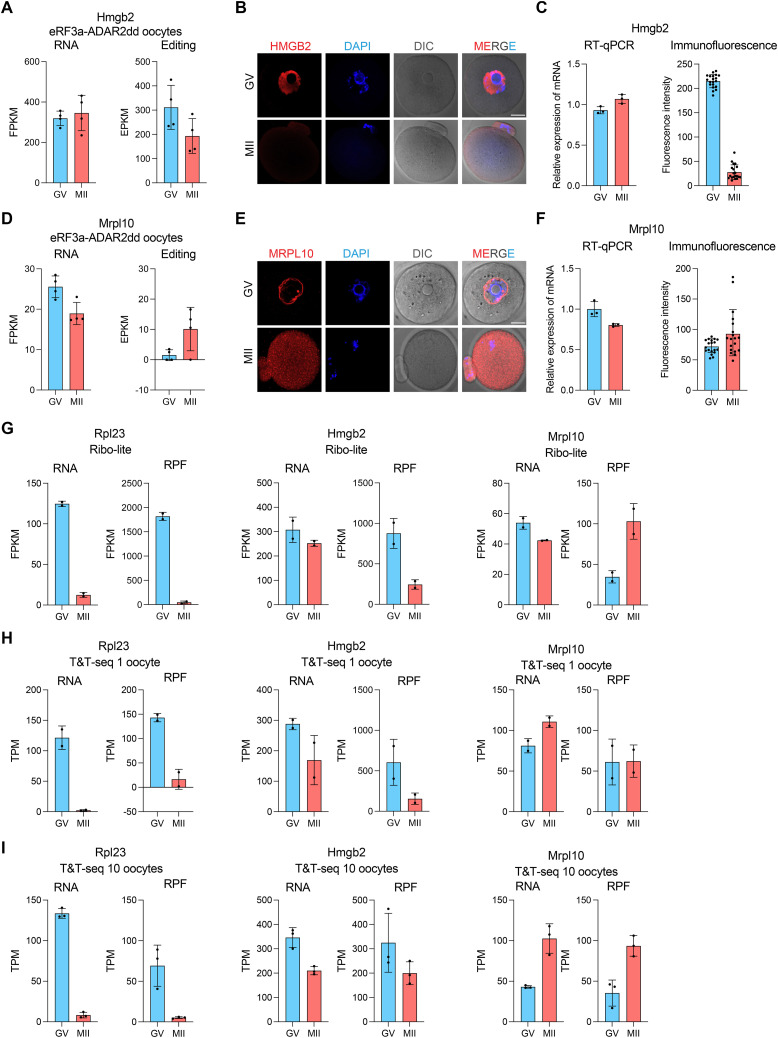
Independent validation of differential RNA translation in mouse oocytes. **(A, B, C)** *Hmgb2* results. **(A)** RNA level and editing level of *Hmgb2* detected in eRF3a-ADAR2dd mRNA-injected oocytes. n = 4. **(B)** Representative immunofluorescence results showing protein level of *Hmgb2* in mouse oocyte. Scale bar, 20 μm. **(C)** RT-qPCR quantification of RNA level (relative to endogenous 18S rRNA, n = 3) and fluorescence intensity quantification of protein level (from immunofluorescence results, n = 20). *P* (RT-qPCR) = 0.0002. *P* (fluorescence intensity) < 0.0001. Statistical significance was calculated using a two-sided unpaired *t* test (default in Prism 9). The experiment was repeated two times independently with similar results. **(D, E, F)** *Mrpl10* results. **(D)** RNA level and editing level of *Mrpl10* detected in eRF3a-ADAR2dd mRNA-injected oocytes. n = 4. **(E)** Representative immunofluorescence results showing protein level of *Mrpl10* in mouse oocyte. Scale bar, 20 μm. **(F)** RT-qPCR quantification of RNA level (relative to endogenous 18S rRNA, n = 3) and fluorescence intensity quantification of protein level (from immunofluorescence results, n = 19). *P* (RT-qPCR) < 0.0001. *P* (fluorescence intensity) = 0.0258. Statistical significance was calculated using a two-sided unpaired *t* test (default in Prism 9). The experiment was repeated two times independently with similar results. **(G, H, I)** RNA level and RPF level of *Rpl23*, *Hmgb2*, and *Mrpl10* measured by the Ribo-lite and T&T-seq approach, respectively. **(G)** By the Ribo-lite approach in a single oocyte. n = 2. **(H)** By the T&T-seq approach in a single oocyte. n = 2. **(I)** By the T&T-seq approach in 10 oocytes. n = 3.

To assess RNA editing, we first compared EPKM values between replicates and found that eRF3a-ADAR2dd mRNA-injected oocytes showed substantially stronger inter-replicate correlation than ADAR2dd-only mRNA-injected oocytes (Figs 2D and E, and S6C and D). Next, we compared the RNA editing profiles from our samples with published single-oocyte translatome data obtained using either the ligation-free, ultra-low-input, and enhanced Ribo-seq (Ribo-lite) (7) or the combined RiboLace and SMARTer-seq approach (T&T-seq) (8). As shown in Figs 2F–I and S7C–J, RNA editing profiles, represented by EPKM, in eRF3a-ADAR2dd mRNA-injected oocytes correlated more strongly with the translatome at both GV and MII stages than those in ADAR2dd-only controls. This observation is consistent with our findings in HEK293T cells, where RNA editing levels in eRF3a-ADAR2dd–expressing cells correlated well with mRNA translation levels. Furthermore, the correlations between eRF3a-ADAR2dd EPKM and the translatome were comparable to those observed between different translatome datasets (Fig S7K–N). To further validate these results, we selected two representative genes (*Hmgb2* and *Mrpl10*) that showed distinct translation dynamics across the two stages. The translation level of *Hmgb2* decreased at the MII stage in RAFTER, Ribo-lite, and T&T-seq 1-oocyte datasets (Fig S8A and G–I [middle panel]), whereas *Mrpl10* exhibited an increased translation level at the MII stage in RAFTER, Ribo-lite, and T&T-seq 10-oocyte datasets (Figs S8D and G) [right panel] and S8I [right panel]). Consistent with these observations, our immune-staining results revealed the corresponding changes in protein abundance, either reduced or increased fluorescence intensity, accompanied by opposite trends in RNA abundance from the GV to the MII stage (Fig S8B, C, E, and F). In addition, we selected one gene (*Rpl23*) that exhibited inconsistent translation levels across three datasets for validation. *Rpl23* showed higher translation level, represented by EPKM, at the MII stage in our data (Fig 2J). In contrast, it showed lower translation level at the MII stage in both the Ribo-lite and T&T-seq datasets (Fig S8G–I, left panel). Our independent validation confirmed higher protein abundance and lower RNA abundance of *Rpl23* at the MII stage (Fig 2K and L). However, we also observed differences in the levels of differential RNA expression and translation level between the two stages in our data compared with the Ribo-lite and T&T-seq data. These discrepancies may be attributed to differences in oocyte preparation strategies. Both Ribo-lite and T&T-seq used in vivo–collected immature and mature oocytes, whereas the oocytes analyzed in our method were matured in vitro. Together, our results demonstrate that, for *Hmgb2*, *Rpl23*, and *Mrpl10*, RAFTER-mediated RNA editing faithfully reflected translation dynamics across developmental stages. Therefore, RAFTER provides a practical and experimentally accessible alternative with simplified procedures for measuring mRNA translation at the single-cell level.

## Discussion

Translation profiling methods such as Ribo-seq and polysome profiling have greatly advanced our understanding of translational regulation, yet ribosome association is not necessarily equal to productive translation. As translation termination generally follows productive decoding of coding sequences, here we developed a novel method, RAFTER that integrates the RNA editing enzyme HyperTRIBE with the translation releasing factor eRF3a, enabling A-to-I editing during the translation termination stage. We demonstrate that RAFTER not only exhibits specificity in detecting protein-coding mRNAs but also shows sensitivity in identifying noncoding RNAs that encode small peptides. Furthermore, without requiring sophisticated experimental procedures, RAFTER achieves measurement accuracy comparable to both ribosome footprinting and polysome profiling. When combined with single-cell sequencing, RAFTER serves as an effective and accessible tool for profiling the translation level during mouse oocyte maturation.

However, several limitations should be considered. First, ribosomes at stop codons may include unrecycled ribosomes, rather than exclusively representing ribosomes that have just completed translation, as reported in previous Ribo-seq studies (32). Second, RAFTER lacks codon-level resolution and therefore cannot directly distinguish individual termination events. Thus, although RAFTER provides a simple and broadly applicable approach for monitoring translation, including in single-cell contexts, it should be considered complementary to existing translation profiling methods rather than a replacement.

## Materials and Methods

### Plasmid construction

To generate an inducible expression plasmid for an eRF3a-ADAR2dd fusion protein, the coding sequence of *eRF3a* (NM_002094.4_cds_NP_002085.3_1) was amplified from cDNA and ligated with the deaminase domain of human ADAR2 with a 25–amino acid flexible linker in between. In addition, an HA-T2A-mCherry cassette was inserted into the C-terminal end of the fusion protein. The resulting eRF3a-25aa linker-ADAR2dd-HA-T2A-mCherry cassette was cloned into the Tet-On 3G vector backbone (courtesy of Dr. Ruijun Tian, Southern University of Science and Technology) to generate the doxycycline-inducible expression plasmid. Nucleotide sequences of fusion proteins are provided in Table S1.

Table S1. Nucleotide sequence of fusion proteins used in this study.

### Cell culture and generation of cell lines

HEK293T cells were obtained from ATCC. Cells were cultured in Dulbecco’s modified Eagle’s medium (DMEM; 11965092; Gibco) supplemented with 10% fetal bovine serum (FBS; SA301.02; Cellmax) and 1% penicillin/streptomycin (15070063; Gibco) at 37°C in a humidified atmosphere containing 5% CO_2_.

RAFTER cell lines were generated using lentiviral transduction. Lentiviruses were prepared using wild-type HEK293T cells at a confluency of about 70%. The target plasmid, pMD2.G plasmid, and psPAX2 plasmid were mixed at a ratio of 2:2:3 with polyethyleneimine (40816ES03; YEASEN) in Opti-MEM (31985062; Gibco). The mixture was incubated for 15 min at room temperature and then added to the cell culture medium. Two days later, the virus-containing medium was collected and mixed with polybrene (40804ES76; YEASEN) for lentiviral transduction. Stably transduced cells were selected using 10 μg/ml blasticidin S (60218ES10; YEASEN) for 5 d. Surviving cells were expanded for storage and further experiments.

### Induction of RNA editing

RAFTER cells were induced with 1 μg/ml doxycycline in DMEM (10% FBS, 1% penicillin/streptomycin) for 48 h, followed by FACS to enrich the successfully induced cells according to mCherry signal. Total RNA was extracted using TRIzol Reagent (15596026; Invitrogen) according to the manufacturer’s protocol. RNA concentration was measured using Equalbit RNA BR Assay Kit (EQ212-01; Vazyme), and 500 ng total RNA was used as input material for the mRNA-seq library construction (12309ES96; YEASEN) following the manufacturer’s protocol.

### Polysome profiling

RAFTER cells were treated with 1 μg/ml doxycycline for 48 h before the experiment. Translating ribosomes were halted by adding cycloheximide (HY-12320; MCE) to the culture medium at a final concentration of 100 μg/ml and incubating the cells at 37°C for 10 min. Cells were then washed with 1× PBS containing 100 μg/ml cycloheximide and harvested by scraping. After centrifugation (300× *g*, 4°C, 5 min), cell pellets were lysed in 300 μl of ice-cold lysis buffer (20 mM HEPES, pH 7.4, 250 mM KCl, 10 mM MgCl_2_, 0.5% NP-40, 5 mM DTT, 100 μg/ml cycloheximide, 1× protease inhibitor cocktail, and 40 U/ml RNase inhibitor). Cell lysates were incubated on ice for 10 min and passed through a 25-gauge needle 10 times. Lysates were centrifuged at 500× *g* for 5 min at 4°C to remove nuclei, followed by centrifugation at 16,363× *g* for 10 min at 4°C to remove debris. The supernatant was loaded onto a 10–50% (wt/vol) sucrose gradient prepared using a BioComp gradient maker. Gradients were centrifuged in an Optima XE-100 with a Beckman SW41 Ti rotor at 222,000*g* for 2 h and 20 min at 4°C and then analyzed using a BioComp gradient fractionator.

### Data processing for RNA-seq

mRNA-seq libraries were sequenced on the Illumina NovaSeq 6000 System in PE150 mode. Sequencing quality was assessed with FastQC (v0.11.9, https://www.bioinformatics.babraham.ac.uk/projects/fastqc/). Adapter sequences were removed using Cutadapt (v1.18) with parameters: -j 40 -m 20 -q 20. The reads were then filtered for those derived from repetitive elements using sequences from the UCSC Genome Browser (https://genome.ucsc.edu/cgi-bin/hgTables) and afterward aligned to the hg38 reference genome with STAR (v2.7.9a). Uniquely mapped reads were quantified using featureCounts (v2.0.1) against GENCODE annotations (v37, Ensembl 103) with the following parameters: -T 24 -s 2 -p -B -C -g gene_name -Q 30. Differential expression analysis was performed using DESeq2 (v1.46.0). Genes with an adjusted *P*-value (padj) <0.05 and an absolute log2 fold change (|LFC|) >1 were considered significantly differentially expressed.

### RNA editing analysis for RNA-seq

After aligning reads to the genome, BAM files were further processed using SAILOR (v1.2.0) (19) to identify A-to-I edit sites across the hg38 reference genome. Edit sites with confidence score larger than 0.65 were retained. The retained edit sites were further filtered based on coverage (coverage ≥10) and mutation frequency (frequency <0.95). Subsequently, for protein-coding genes, sites annotated within the 5′ UTR, CDS, or 3′ UTR were extracted for further analysis. For noncoding genes, sites annotated within the exon were extracted for further analysis. To characterize the distribution of RNA editing activity around translation start and stop sites, editing sites located within −2,800 nt of the start and +2,800 nt of the stop codon of the longest protein-coding transcript for each gene were extracted. The region was further divided into 40 equally sized bins, and the number of editing sites within each bin was counted to generate a positional density profile. To obtain a smooth representation of the editing distribution, cubic spline interpolation was applied to the binned counts and the resulting continuous density curves were plotted.

Transcripts per kilobase million (TPM) for each gene was calculated by normalizing read counts for sequencing depth and gene length. Genes with a TPM >1 in the sample were included in ES analyses. To compare editing at the gene level between the cells expressing eRF3a-ADAR2dd protein and ADAR2dd-only, the ES for each gene was calculated as the ratio between the number of edited reads and that of total reads mapped to the gene. To identify genes that were significantly edited, Fisher’s exact test was applied to examine the statistical significance of the ES. The maximum *P*-value across the comparison results for all replicates was used as the final *P*-value for each gene. Then, the final *P*-value was adjusted for multiple comparisons using the Benjamini and Hochberg procedure. Genes with an FDR <0.05 and an LFC of ES >1 were considered as significantly edited (Table S2).

Table S2. The quantification of editing levels in RAFTER HEK293T cells.

To more accurately reflect the association of fusion proteins with RNAs, we introduced EPKM as follows:

$$\text{EPKM}=\text{edits}\times \frac{10^{3}}{\text{window}}\times \frac{10^{6}}{\text{total}\;\text{edits}}$$

The longest protein-coding transcripts of each gene were used for analysis. The window was limited from –100 to +2,000 nt of the start codon and from –1,000 to +200 nt of the stop codon. Specifically, for a given gene, the window size was defined as: min{5′UTR length, 100} + min{CDS length, 3,000} + min{3′UTR length, 200}. Edits represented the total number of editing events detected within the defined window of the respective gene. Total edits represented the sum of editing events across all genes of respective samples. Only genes with FPKM >0 were considered.

### In vitro transcription

The EGFP-UTR cassette was cloned into the pcDNA6 plasmid, where a specific edit site for eRF3a-ADAR2dd was in the UTR region. The resulting plasmid was linearized using NotI-HF and AgeI-HF (R3552; NEB). EGFP-UTR mRNAs were in vitro transcribed using the T7 High Yield RNA Synthesis Kit for Co-transcription (10673ES50; YEASEN) from the linearized plasmid template. To generate mRNAs without the 5′ cap, the GAG component was substituted with RNase-free H_2_O. In vitro–transcribed mRNAs were purified using RNA Clean & Concentrator-25 (R1018; ZYMO) and polyadenylated by *E.coli* poly(A) polymerase (14801ES60; YEASEN). To generate mRNAs without a poly(A) tail, the polyadenylation step was omitted. After polyadenylation, mRNAs were purified using RNA Clean & Concentrator-5 (R1014; ZYMO). The nucleotide sequence of EGFP-UTR is provided in Table S3.

Table S3. Nucleotide sequence of the reporter mRNA used in this study.

### mRNA transfection and RNA editing quantification

RAFTER cells were seeded into 6-well plates at 0.4 million cells per well for 24 h. Then, 1 μg/ml doxycycline was supplemented into the culture medium for 36 h. Next, 200 ng of mRNA was transfected into cells using mRNA Transfection Reagent (40809ES01; YEASEN). Two hours after transfection, the culture medium containing mRNAs was replenished with fresh culture medium containing 1 μg/ml doxycycline. Cells were collected for flow-cytometry analysis and RNA extraction after another 10 h. Total RNA was extracted using TRIzol Reagent according to the manufacturer’s protocol. RNA concentration was measured using Equalbit RNA BR Assay Kit, and 2 μg total RNA was used for cDNA synthesis using Hifair III 1st Strand cDNA Synthesis Kit (gDNA digester plus). cDNA synthesis was conducted using random N6 primers only. Amplicons containing the edit site for Sanger sequencing were then amplified (P515; Vazyme) using primers shown in Table S4. A-to-I editing level was quantified using the EditR package (33). Primers for RT-qPCR are shown in Table S4.

Table S4. Oligos used in this study.

### Mouse oocytes preparation and injection

All animal procedures were approved by the Animal Research Committee of Nanjing Medical University and performed in accordance with Institutional guidelines. Fully grown GV-stage oocytes were collected from 4-wk-old female ICR mice. Before microinjection, oocytes were cultured in M2 medium (M7167; Sigma-Aldrich) with 2 μM milrinone (M4659; Sigma-Aldrich) to prevent germinal vesicle breakdown. mRNAs encoding eRF3a-ADAR2dd or ADAR2dd-only were in vitro transcribed (HiScribe T7 ARCA mRNA Kit with tailing, E2060S; NEB) and microinjected into oocytes (∼5–10 pl at 500 ng/μl) using an Eppendorf TransferMan 4r micromanipulator. After injection, oocytes were cultured for 24 h under milrinone arrest to obtain GV-stage samples. For MII-stage samples, oocytes were cultured with milrinone for 12 h and then released into milrinone-free medium for an additional 12 h.

### Immunofluorescence and confocal microscopy

For immunofluorescence staining, oocytes were fixed in 4% paraformaldehyde (in 1× PBS) for 30 min at room temperature, permeabilized in 0.5% Triton X-100 (in 1× PBS) for 20 min and blocked in 1% bovine serum albumin (in 1× PBS). Subsequently, the oocytes were incubated overnight at 4°C with primary antibodies diluted in blocking solution, followed by three washes in PBS and incubation with secondary antibodies for 1 h at room temperature. Nuclei were counterstained with DAPI (5 μg/ml) for 15 min. After final washing, the oocytes were mounted on glass slides using SlowFade Gold Antifade Reagent (Life Technologies) and imaged with a Zeiss LSM900 confocal microscope. Antibody details are provided in Table S5. Semiquantitative analysis of fluorescence signals was performed using ImageJ software.

Table S5. Antibodies used in this study.

### Single-oocyte RNA-seq

We constructed the single-oocyte RNA-seq libraries following the SMART-seq2 protocol (34). Briefly, each oocyte was lysed in 2 μl of lysis buffer (0.2% Triton X-100 and 40 U/ml Recombinant RNase Inhibitor [2313A; Takara]) and then incubated in 10 μl Oligo(dT) buffer (1 μl 10 mM dNTP, 1 μl 10 μM oligo(dT) Primer, 8 μl nuclease-free water) at 72°C for 3 min and immediately placed on ice for 2 min. First-strand cDNA synthesis was performed by adding 4 μl of 5× RT buffer and 4 μl reverse transcriptase enzyme (RK20310; ABclonal), followed by cDNA amplification using amplification module PCR mix (1 μl PCR primer and 29 μl PCR master mix) for 16 cycles. The resulting library was purified using 1× DNA Clean Beads (N411-01; Vazyme). Tagmentation was carried out using Tn5 Enzyme Mix and PCR Mix (RK20237; ABclonal) to construct sequencing libraries from the amplified cDNA according to the manufacturer’s protocol. Final DNA library amplification was performed for 15 cycles using 25 μl PCR Mix and 5 μl amplification primers (10 μM), followed by purification with 0.6× and 0.15× DNA Clean Beads (N411-01; Vazyme). Library quality was assessed using a PerkinElmer LabChip GX Touch system and sequencing was performed on the Illumina NovaSeq 6000 platform in PE150 mode.

### RNA isolation and real-time RT-qPCR

For each sample replicate, five oocytes were collected and lysed in 2 μl of lysis buffer (0.2% Triton X-100 and 40 U/ml Recombinant RNase Inhibitor [2313A; Takara]), and cDNA synthesis was followed by reverse transcription with PrimerScript II Reverse Transcriptase (2690A; Takara) according to the manufacturer’s protocol. RT-qPCR analysis was performed using Power SYBR Green PCR Master Mix (Q712-02; Vazyme) and an Applied Biosystems QuantStudio 5 Real-Time PCR system using the primers listed in Table S4. Relative mRNA expression levels were calculated relative to the levels of endogenous 18S rRNA (used as a housekeeping gene), and each RT-qPCR experimental reaction was performed in triplicate.

### Data processing for single-oocyte RNA-seq

Data processing for single-oocyte RNA-seq was performed similarly to the bulk RNA-seq analysis. Read alignment was conducted according to the GENCODE annotation (vM34, GRCm39), and SNP information was obtained from the dbSNP dataset (GVF_000001635.24, VCFv4.0). Strand orientation for each read was determined based on the GENCODE annotation. The resulting BAM files containing reads mapped to genes on the positive and negative strands were used as inputs to SAILOR (v1.2.0) for determining strand-specific A-to-I edit sites. For replicate comparisons, genes with EPKM or FPKM = 0 in all compared datasets were excluded. For the remaining genes, log2(EPKM+1) values were calculated and *Pearson* correlation coefficients (R) were determined. Correlation plots were generated using the pandas.plotting module (v2.2.3).

### Comparative analysis between RAFTER and analogous methods

Mean EPKM and FPKM were calculated across biological replicates within each group in our data. Ribo-seq data of HEK293T cells were obtained from Shu et al., 2025 (28 *Preprint*). Polysome-seq data of HEK293T cells were obtained from Sun et al., 2025 (20). The processed RPF and RNA-seq data of the Ribo-lite approach were obtained from the Table S1 of Xiong et al., 2022 (7). The processed transcriptome and translatome data of the T&T-seq approach were obtained from the Supplementary Data 1 of Hu et al., 2022 (8). To assess the relationship between RAFTER and analogous methods, genes with either EPKM or FPKM/TPM >0 in all compared datasets were included. For RNA level comparison, respective values were transformed as log2(value+0.01) before calculating the *Pearson* correlation coefficient (R). For translation-level comparison, respective values were transformed as log2(value+1) before calculating the *Pearson* correlation coefficient (R).

## Supplementary Material

Reviewer comments

## Data Availability

The raw sequence data reported in this article have been deposited in the National Genomics Data Center, which are publicly accessible at https://ngdc.cncb.ac.cn/gsa/browse/CRA033778 and https://ngdc.cncb.ac.cn/gsa-human/browse/HRA014848. Source data are provided with this article.
