# Supplementary material for: RAFTER: a releasing factor tethered RNA editing system for quantifying mRNA translation
Source: Life Sci Alliance. 2026 Jul 23;9(10):e202603651. doi: 10.26508/lsa.202603651 (PMC13395895; doi:10.26508/lsa.202603651)
Supplement: Reviewer comments [file LSA-2026-03651_review_history.pdf]

RAFTER: a releasing factor tethered RNA editing system for quantifying mRNA translation

Zhiyuan Sun, Peng Dong, Huanyu Yan, Xiaozhen Wen, Yanping Li, Feifei Jiang, Liang Fang, Xi Wang, and Wei Chen
DOI: https://doi.org/10.26508/lsa.202603651

Corresponding author(s): Wei Chen, Southern University of Science and Technology and Xi Wang, Nanjing Medical University

Review Timeline:

Submission Date:	2026-02-02
Editorial Decision:	2026-02-03
Revision Received:	2026-05-24
Editorial Decision:	2026-06-23
Revision Received:	2026-07-02
Accepted:	2026-07-07

Scientific Editor: Tim Fessenden

Transaction Report:

(Note: With the exception of the correction of typographical or spelling errors that could be a source of ambiguity, letters and reports are not edited. The original formatting of letters and referee reports may not be reflected in this compilation.)

Please note that the manuscript was previously reviewed at another journal and the reports were taken into account in the decision-making process at Life Science Alliance.

Reviewer 1:

Report for Author:
This manuscript introduces RAFTER, a translation-termination-tethered RNA editing approach to infer mRNA translation efficiency. Conceptually, tethering ADAR to eRF3a is elegant and biologically well-motivated, and the method convincingly reports bona fide translation rather than mere ribosome association. The authors provide strong validation in HEK293T cells and an impressive application to mouse oocytes, where existing methods are technically challenging.

Overall, the work is novel, technically sound, and well validated, with clear advantages over polysome profiling and Ribo-seq in terms of accessibility and scalability. With some clarifications, this manuscript should be suitable for publication in MSB.

1. The manuscript alternates between describing RAFTER as measuring "translation rate", "translational efficiency" and "translation level". Please make it consistent and describe what RAFTER editing truly reflects.

2. The eRF3a-ADAR2dd overexpression may potentially perturb the translation as eRF3a is a core translation termination factor. Overexpression or fusion could, in principle, alter termination dynamics or ribosome recycling. The authors should at least address this caveat and if possible examine whether global translation or cell growth was affected.

3. Start-codon editing peak is intriguing but speculative. Present this as a hypothesis rather than a conclusion. The authors may consider citing some relevant papers to support the speculations or possible explanations.

4. EPKM is central but can be hard to understand as it may be the first time to be defined. I also wonder if the authors have considered the challenge of mapping reads that may have multiple editing sites. These hyper-editing events, which indicate the strongest signal, should not be missed due to mapping challenges. There are previously developed methods addressing this (e.g. https://pubmed.ncbi.nlm.nih.gov/25158696/).

Reviewer 2:

Report for Author:
In this manuscript, Sun et al. reported an approach called RAFTER, by fusing ADAR to eRF3, to measure translation efficiency. The rationale assumes that eRF3 binding to mRNA acts

as a proxy for ribosome occupancy. The authors compared RAFTER with standard Ribo-seq and Polysome-seq but the correlation was suboptimal. Additionally, the authors claimed that RAFTER can be applied to single cell analysis of translatome. However, no new findings were reported on oocyte development from germinal vesicle to metaphase II.

The manuscript was written in a very brief manner, and the advantage of RAFTER is not convincing. From the technological perspective, ADAR has been employed for multiple purposes and eIF3a-ADAR is just a small increment. From the biological perspective, whether eRF3 association can be used to assess translation efficiency is still debatable. From the practical perspective, the requirement of transfection of eRF3a-ADAR limits its broad application. The current analysis of RAFTER is superficial, and many conclusions lack orthogonal validation. Overall, the manuscript is short of exciting findings and the impact is limited.

Main concerns

1. The choice of eRF3 over eRF1 in the RAFTER design is questionable. Many studies have fused tags to eRF1 and showed minimal perturbation (e..g., https://doi.org/10.1101/2024.09.01.610654). In eukaryotic cells, eRF3 is not a major termination factor. At least the authors should give eRF1 a try in the very beginning.

2. It has been reported that termination efficiency is different among three stop codons (i.e., UAA, UAG, UGA). Have the authors analyzed mRNAs stratified by stop codons? It is conceivable that different stop codons have distinct dwell time for eRFs and therefore affect the modification occurrence. This result is expected to complicate the data interpretation about translation efficiency.

3. Only 1,799 annotated protein-coding genes were identified as editing targets. Why is the number so small? This is way fewer than regular Ribo-seq and raises the question of the sensitivity of RAFTER.

4. How could the ADAR2dd-only control also show stop codon accumulation? This raises the question of the specificity of RAFTER.

5. The accumulation of editing at start codons is surprising and worth in-depth analysis. Unfortunately, the authors did not pursue further if such phenomenon is true. Notably, the relative positions of edits are different between stop and start codons. While stop codons are associated with upstream peaks, start codons are accompanied with downstream edits. This cannot be explained by simple start and stop codon interaction.

6. The correlation shown in Fig. 1h-k is poor and questions whether RAFTER could be truly used to evaluate translation efficiency. It is also troubling that the negative control ADAR2dd-only also showed position correlation.

7. The reporter assay shown in Fig. 2a-b is informative but not quantitative. Ideally, positive cap&tail reporters with different translation efficiency should be included. For instance, inserting secondary structures in 5'UTR, introducing non-optimal codons in CDS, etc.

8. The results obtained from oocytes are not impressive, and appear to be less informative than Ribo-lite and T&T-seq. In fact, RAFTER requires injection of exogenous eRF3a-ADAR2dd, which is impractical in many situations such as in vivo samples. This disadvantage limits its broad application, not to mention the possibility of artifacts.

Reviewer 3:

Report for Author:
In this manuscript, the authors developed RAFTER, a novel tool to measure mRNA translation by fusing the release factor eRF3a with the deaminase domain of ADAR2. The authors demonstrate that RAFTER can detect translated mRNAs through A-to-I editing at termination sites and apply this method to single mouse oocytes. The concept of using release factor with deaminase to mark translated mRNAs is interesting and could be consistent with previous Ribo-lite and T&T-seq data. However, this reviewer found critical concerns about the novelty of the methodology, robustness of the technique itself, and the biological significance. This reviewer suggests that a more specific journal may be more suitable for this manuscript.

Major comments
1. First and foremost, this method is essentially the same concept as (Ribo-)STAMP and (Ribo-)TRIBE, published in earlier papers. The difference is only in the usage of eRF3a for tagging. The authors should compare their methods with those earlier methods directly side-by-side and demonstrate that eRF3a tagging outperforms others.

2. The advantage of RAFTER over Ribo-Lite and T&T-seq is unclear from the results. Both methods have already achieved single-oocyte resolution. The authors should show the clear practical benefit of using RAFTER instead of established methods.

3. Given the data shown in Fig. 1f in the metagene plot, free ADAR2dd, without tagging to eRF3a, could label the RNA around the stop codon. This suggested that 1) ADAR2dd may

have the sequence preference, that 2) this may match with the natural sequence context around the stop codon, and that 3) ultimately, off-target effects may ruin the sensitivity. This condition is not appropriate for experiments.
3a. The authors should test if such an intrinsic sequence preference and off-target effect exist in ADAR2dd.
3b. The authors should test if such a sequence preference could be minimized by the expression level control (e.g., low-level expression of the construct).
3c. If these could not be solved, the authors should avoid using eRF3a for translational profiling.
3d. ADAR2dd is the catalytic domain only, but previous reports (DOI: 10.1016/j.cell.2016.03.007) show that it still prefers dsRNA structures over ssRNA. The authors should analyze whether editing efficiency correlates with local RNA secondary structure.

4. In Figure 1g, the authors observed A-to-I editing peaks around the start codon and discuss that this peak reflects the mRNA closed-loop structure. However, this reviewer has several concerns:
4a. This may also stem from the sequence preference, as free ADAR2dd provided the editing enrichment at the same locus.
4b. Editing also occurs at ~2000 nt from the start codon. Considering the additional RNA region in the 5' UTR of each mRNA, this seems physically too far from the cap structure. If a closed-loop structure by cap-poly(A) interaction affects labeling in that region, labeling should occur in the cap-proximal region. However, the authors did not observe frequent labeling at the 5' UTR. The same conflict should apply to the 3' UTR. The authors pointed out the "additional" mechanism for the closed loop. However, the evidence shown in this manuscript did not support this.
4c. The authors should use a reporter assay, as in Fig. 2a-b, to test the editing site around the start codon. The presence and absence of a cap and polyA tail in the in vitro transcribed reporter may be helpful for the discussion of the closed-loop model.
4d. As an alternative explanation to point 4a above, the weird signal at the start codon may come from the stop codons of uORFs. The authors should carefully survey this possibility.

5. The authors should show a coverage plot across 5'UTR-CDS-3'UTR. The current metagene plot focuses on start and stop codon regions, but editing may also occur within CDS, considering the labeling at the start codon region. This analysis would clarify whether editing is truly enriched at the stop codon or distributed throughout the CDS. If editing is also distributed within CDS, the authors should visualize metagene profiles around near-cognate stop codons and at low-optimality codons to assess the possibility that eRF3a

recognizes near-cognate codons or associates with ribosomes at suboptimal codons. Such a misrecognition has been reported in earlier reports (DOI: 10.1111/febs.14709 and DOI: 10.1093/nar/gkz710). Also, the authors should test whether the CDS/UTR length, codon contents, and others may affect the editing rates.

6. In Fig. 1d, the authors claimed that ncRNA translation could be detected. The authors should show the editing rate along the ncRNAs and short ORFs. The editing sites should correspond to the stop codon of the short ORFs in ncRNAs.

7. ADAR2dd-only control shows Pearson R = 0.3 in HEK293T and R = 0.47-0.49 in oocytes (Fig. 1h, 1j, Fig. 2f, 2h). This is unexpectedly high for a negative control. The authors should discuss the reason for this high correlation. This reviewer suspects that high expression of ADAR2dd may edit exposed mRNA regions, resulting in increased off-target labeling (as suggested in the point above). Generally, this potential off-target correlation should be correlated with RNA abundance in cells. RNA abundance and Ribo-seq/polyosome-seq should show good correlation. Thus, the ADAR2dd-only may show a medium correlation (Fig. 1h, 1j, Fig. 2f, 2h). Again, the authors should revisit their conditions to minimize the off-target effects. The authors may want to investigate the mitochondria genome-encoded mRNAs, since they may serve as a good negative control.

8. Related to the point above, this manuscript did not report translation efficiency (i.e., translation rate per mRNA). In theory, using RNA abundance and editing rate, the authors could analyze this. This should be an important aspect in the field of translation regulation.

9. The overexpression of termination factor (as a fusion with ADAR2d) may affect endogenous translation (or specifically termination). The authors should examine whether eRF3a-ADAR2dd fusion affects global translation level (e.g., FUNCAT, puromycin incorporation, or sucrose density gradient).

10. In Lines 163-175, the authors summarized the published data as "increased" or "decreased". However, some of the panels did not follow this tendency. The authors should not generalize the data, ignoring the contradictions.

Minor comments

1. Figure 2a, 2b: "bp" should be "base" or "nucleotide" since it refers to single-stranded RNA.

2. Line 199-206: The source of the ADAR2dd sequence is not specified. Also, the information on the plasmid backbone was not shown. The authors should clarify these.

3. This manuscript has a lot of errors in figure citations, including:
Line 111: Fig. 1g should be Fig. 1h
Line 112: Fig. 1i should be Fig. 1j
Line 115: Fig. 1k should be removed
Line 128: Supplementary Figure 3b and 3c should be Supplementary Fig. 3c and 3d
Line 147: Supplementary Fig. 5a and 5b should be Supplementary Fig. 4a and 4b
Line 153-154: Supplementary Figure 4a and 4b should be Supplementary Figure 4c and 4d
Line 167: "the left panel" should be the "right panel"
Line 175: "the left panel" should be inserted after Supplementary Fig. 6g-i

Also, Supplementary Fig. 4e and 4f were not cited in the manuscript.

4. This manuscript does not have a subheading for Introduction, Results, and Discussion. This should be included.

5. Regarding the discussion in Line 47, there are a couple of reports of single-cell Ribo-Seq and several others for low-input tailored Ribo-Seq. This discussion should be reconsidered.

6. Lines 44-45 and Lines 51-52, the same discussion is repeated. The authors should omit one.

7. Lines 44-45 and Lines 51-52, the authors claimed that ribosome association may not always mean active translation. This reviewer agrees with this; however, the authors should cite key references to support this.

8. Lines 44-45 and Lines 51-52, the ribosome on the stop codon may consist of unrecycled ribosomes (reported in many papers with Ribo-seq). Targeting the stop codon itself may not overcome this issue.

9. Line 54, the authors should add more rationale for why targeting the termination stage may overcome the issues in the earlier methods.

10. Line 120, this reviewer would say "an endogenous A site" should be "an endogenous site".

11. In Fig. 1-2 and Supplementary Figs. 2, 4, 5, scatter plots may have diagonal lines; however, the detail of this was not explained in the legends. This reviewer guesses that these are regression lines, but some of the lines may not look like this (dranw far from the expectation).

February 3, 2026

Re: Life Science Alliance manuscript #LSA-2026-03651-T

Prof Wei Chen
Southern University of Science and Technology
Department of Biology
1088 Xueyuan Avenue
Shenzhen, Guangdong 518005
China

Dear Dr. Chen,

Thank you for transferring your manuscript entitled "RAFTER: a releasing factor tethered RNA editing system for quantifying mRNA translation" to Life Science Alliance. In accordance with our offer to consider this work, and our subsequent correspondence with you on the previous reviewer requests, we invite you to submit a revised manuscript addressing the following concerns:

- Address all points raised by Reviewer 1 with changes to the text, in particular by clearer definition of EPKM with potential drawbacks as noted by this reviewer.
- Discuss in the results and discussion sections the observations made with the ADARdd only control, in particular related to Fig 1F, as noted by Reviewer 2 in points 4 and 6 and Reviewer 3 in point 3.
- Include analysis of different stop codons or, at a minimum, adjust the text to remark that these may skew the results, per Reviewer 2 in point 2.
- Temper the claims on start codon editing per Reviewer 3 point 4.

Upon receipt of a revised manuscript according to the above points, we will return this work to the original Reviewer 1 and Reviewer 3 for their evaluation. A point-by-point rebuttal letter should include responses to all reviewer comments in some form, however implementing additional revisions besides those noted above is left to your discretion.

In case you have any questions I would be happy to discuss the revision in more detail via email or phone/videoconferencing. Please let me know which option you prefer, if any.

To upload the revised version of your manuscript, please log in to your account: https://lsa.msubmit.net/cgi-bin/main.plex

You will be guided to complete the submission of your revised manuscript and to fill in all necessary information. Please get in touch in case you do not know or remember your login name.

The typical timeframe for revisions is three months. Please note that papers are generally considered through only one revision cycle, so strong support from the referees on the revised version is needed for acceptance.

While you are revising your manuscript, please also attend to the below editorial points to help expedite the publication of your manuscript. Please direct any editorial questions to the journal office.

Thank you for this interesting contribution to Life Science Alliance. We are looking forward to receiving your revised manuscript.

Sincerely,

Tim Fessenden
Scientific Editor
Life Science Alliance

A. THESE ITEMS ARE REQUIRED FOR REVISIONS

-- A letter addressing the reviewers' comments point by point.

-- An editable version of the final text (.DOC or .DOCX) is needed for copyediting (no PDFs).

-- High-resolution figure, supplementary figure and video files uploaded as individual files: See our detailed guidelines for preparing your production-ready images, https://www.life-science-alliance.org/authors

-- Summary blurb (enter in submission system): A short text summarizing in a single sentence the study (max. 200 characters including spaces). This text is used in conjunction with the titles of papers, hence should be informative and complementary to the title and running title. It should describe the context and significance of the findings for a general readership; it should be written in the present tense and refer to the work in the third person. Author names should not be mentioned.

-- By submitting a revision, you attest that you are aware of our payment policies found here: https://www.life-science-alliance.org/copyright-license-fee

B. MANUSCRIPT ORGANIZATION AND FORMATTING:

Full guidelines are available on our Instructions for Authors page, https://www.life-science-alliance.org/authors

We encourage our authors to provide original source data, particularly uncropped/-processed electrophoretic blots and spreadsheets for the main figures of the manuscript. If you would like to add source data, we would welcome one PDF/Excel-file per figure for this information. These files will be linked as supplementary "Source Data" files.

IMPORTANT: It is Life Science Alliance policy that if requested, original data images must be made available. Failure to provide original images upon request will result in unavoidable delays in publication. Please ensure that you have access to all original microscopy and blot data images before submitting your revision.

Reviewer #1:

This manuscript introduces RAFTER, a translation-termination-tethered RNA editing approach to infer mRNA translation efficiency. Conceptually, tethering ADAR to eRF3a is elegant and biologically well-motivated, and the method convincingly reports bona fide translation rather than mere ribosome association. The authors provide strong validation in HEK293T cells and an impressive application to mouse oocytes, where existing methods are technically challenging. Overall, the work is novel, technically sound, and well validated, with clear advantages over polysome profiling and Ribo-seq in terms of accessibility and scalability. With some clarifications, this manuscript should be suitable for publication in MSB.

1. The manuscript alternates between describing RAFTER as measuring "translation rate", "translational efficiency" and "translation level". Please make it consistent and describe what RAFTER editing truly reflects.

Author Response: We thank the reviewer for this critical comment. We have now standardized the text, using the term "translation level" throughout the manuscript. As RAFTER utilizes a translation termination-dependent deaminase activity to mark translated transcripts, this metric reflects the cumulative translational activity associated with a given mRNA transcript.

2. The eRF3a-ADAR2dd overexpression may potentially perturb the translation as eRF3a is a core translation termination factor. Overexpression or fusion could, in principle, alter termination dynamics or ribosome recycling. The authors should at least address this caveat and if possible examine whether global translation or cell growth was affected.

Author Response: We appreciate the reviewer's concern regarding the potential for eRF3a-ADAR2dd to interfere with endogenous translation machinery. To address this, we systematically evaluated the impact of eRF3a-ADAR2dd overexpression. First, we performed RNA-seq to compare the transcriptomes of cells with TetON-induced expression of ADAR2dd or eRF3a-ADAR2dd against wild-type (WT) cells. As shown in Figure R1a and R1b, the induction of either construct resulted in minimal transcriptomic changes from WT. Notably, a direct comparison between eRF3a-ADAR2dd and ADAR2dd-expressing cells also revealed minimal transcriptomic changes (Fig. R1c), suggesting that the fusion protein expressed at the levels used in our studies does not substantially perturb homeostatic gene expression. Second, to evaluate the effect of eRF3a-ADAR2dd overexpression to the global translation, we performed polysome profiling. The profiles for ADAR2dd-expressing and eRF3a-ADAR2dd-expressing cells are nearly identical (Figure R1d). These data collectively demonstrate that eRF3a-ADAR2dd expression, at the level in our study, does not impair cellular homeostasis or global translation.

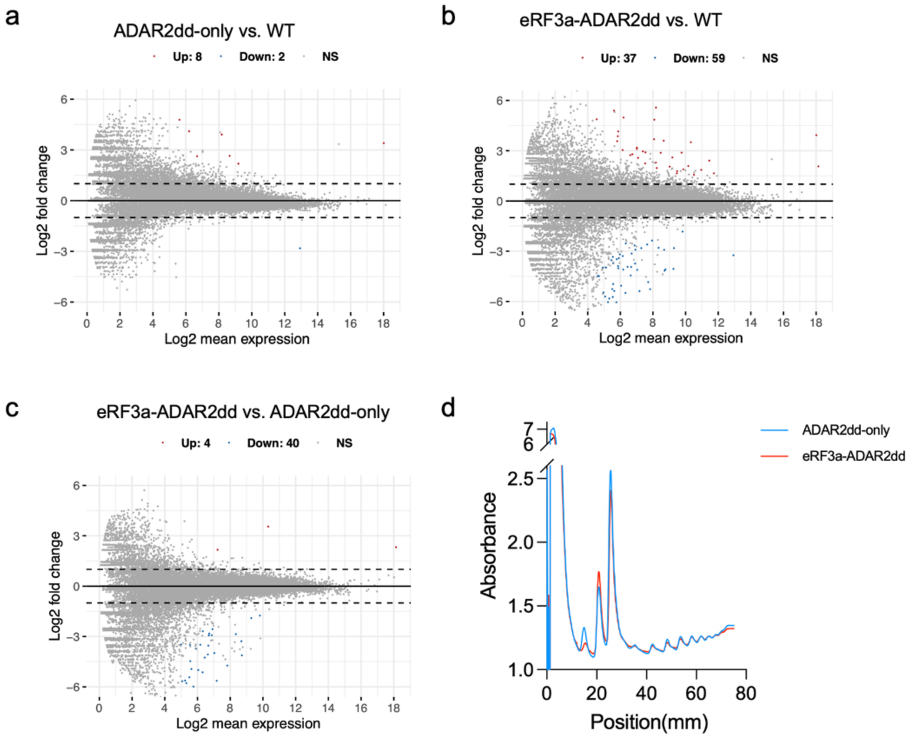

Figure R1. a-c, MA plots showing differential gene expression profiles between the indicated samples. Comparisons are specified at the top of each panel. **a**, ADAR2dd-only cells vs. wild-type (WT) cells. **b**, eRF3a-ADAR2dd cells vs. WT cells. **c**, eRF3a-ADAR2dd cells vs. ADAR2dd-only cells. **d**, Polysome profiling showing the distribution of RNAs associated with free fractions (non-ribosome-bound), 40S ribosomal subunits, 60S ribosomal subunits, 80S monosomes, and polysomes in ADAR2dd-only cells (blue line) and eRF3a-ADAR2dd cells (red line).

3. Start-codon editing peak is intriguing but speculative. Present this as a hypothesis rather than a conclusion. The authors may consider citing some relevant papers to support the speculations or possible explanations.

Author Response: We thank the reviewer for this insightful suggestion. We agree that our explanation for the observed start-codon editing peaks is currently speculative and should be presented as a hypothesis rather than a definitive conclusion. Accordingly, we have revised the text to soften the interpretation and added relevant references (Pages 5-6, Lines 97-100). Direct experimental validation of start-stop codon proximity is beyond the scope of the current study. Nevertheless, we are currently collaborating with colleagues to investigate whether actively translating mRNAs can exhibit spatial proximity between start and stop codons using cryo-ET-based approaches.

4. EPKM is central but can be hard to understand as it may be the first time to be defined. I also wonder if the authors have considered the challenge of mapping reads that may have multiple editing sites. These hyper-editing events, which indicate the strongest signal, should not be missed due to mapping challenges. There are previously developed methods addressing this (e.g. https://pubmed.ncbi.nlm.nih.gov/25158696/).

Author Response: We thank the reviewer for these insightful suggestions.

Regarding EPKM (edited reads per kilobase per million mapped reads), this metric was originally introduced by Brannan et al. (PMID: 33963355), where the combined length of the CDS and 3′ UTR was used to normalize editing events. In our study, because eRF3a-ADAR2dd-mediated editing was predominantly enriched within approximately -100 to +2000 nt relative to the start codon and -1000 to +200 nt relative to the stop codon, we instead used these effective editing regions as the normalization

window (Fig. R2a). Specifically, for a given gene X, the window size was defined as: min{5′UTR length, 100} + min{CDS length, 3000} + min{3′UTR length, 200}. “Edits” denotes the total number of editing events detected within the defined window of gene X, whereas “Total Edits” represents the sum of editing events across all genes within the sample. We have expanded the description of this metric in the revised manuscript on Page 6, Lines 102-107.

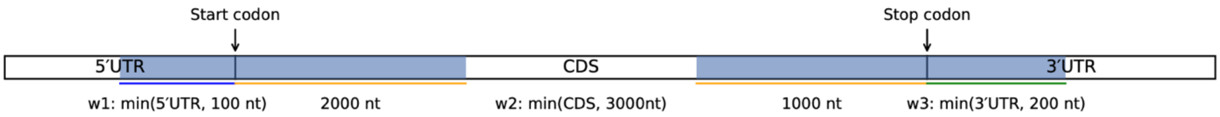

$$\mathrm{EPKM} = \mathrm{edits} \times \frac{10^3}{\mathrm{window}} \times \frac{10^6}{\mathrm{total\ edits}} (window = w1 + w2 + w3)$$

Figure R2. Schematic illustration of EPKM calculation.

Regarding hyper-editing events, we investigated whether our current pipeline might inadvertently exclude highly edited reads during alignment. Following the reviewer’s suggestion, we implemented a hyper-editing-aware re-mapping strategy for previously unmapped reads by computationally converting all adenosines to guanosines (A-to-G) in both the reads and the reference genome, as described by Porath et al. (PMID: 25158696). Using this strategy, only seven genes contain hyper-editing events. The corresponding editing levels, quantified by EPKM before and after the re-mapping procedure, are summarized in Table R1. Notably, our original analysis pipeline already excluded highly repetitive regions, which are the primary genomic contexts reported by Porath et al. to harbor extensive hyper-editing. Furthermore, only 7 out of 14,310 analyzed genes were affected by the re-mapping procedure. Therefore, we believe that hyper-editing events have minimal impact on our overall analysis and conclusions.

Gene names	Original EPKM (Rep.1)	New EPKM (Rep.1)	Original EPKM (Rep. 2)	New EPKM (Rep.2)
CEBPZOS	687.094	897.039	299.111	299.111
MRPL30	671.066	952.111	617.227	802.994
PRR11	188.725	268.188	211.015	401.274
RPL37A	1008.868	2517.784	1072.385	2080.610
BPNT1	316.249	316.249	319.019	341.806
PSMB2	496.277	496.277	523.689	674.055
SDHAF4	407.410	407.410	364.849	425.657

Table R1. Summary of EPKM values for hyper-edited genes before and after A-to-G re-mapping.

Reviewer #2:

In this manuscript, Sun et al. reported an approach called RAFTER, by fusing ADAR to eRF3, to measure translation efficiency. The rationale assumes that eRF3 binding to mRNA acts as a proxy for ribosome occupancy. The authors compared RAFTER with standard Ribo-seq and Polysome-seq but the correlation was suboptimal. Additionally, the authors claimed that RAFTER can be applied to single cell analysis of translatome. However, no new findings were reported on oocyte development from germinal vesicle to metaphase II.

The manuscript was written in a very brief manner, and the advantage of RAFTER is not convincing. From the technological perspective, ADAR has been employed for multiple purposes and eIF3a-ADAR is just a small increment. From the biological perspective, whether eRF3 association can be used to assess translation efficiency is still debatable. From the practical perspective, the requirement of transfection of eRF3a-ADAR limits its broad application. The current analysis of RAFTER is superficial,

and many conclusions lack orthogonal validation. Overall, the manuscript is short of exciting findings and the impact is limited.

Main concerns

1. The choice of eRF3 over eRF1 in the RAFTER design is questionable. Many studies have fused tags to eRF1 and showed minimal perturbation (e..g., https://doi.org/10.1101/2024.09.01.610654). In eukaryotic cells, eRF3 is not a major termination factor. At least the authors should give eRF1 a try in the very beginning.

Author Response: We thank the reviewer for these thoughtful suggestions.

Regarding the choice of eRF3 over eRF1, we note that the study suggested by the reviewer fused a relatively small streptavidin-binding peptide (38 aa, ~4.3 kDa) to eRF1. In contrast, the ADAR2 deaminase domain (ADAR2dd) used in our system comprises 385 amino acids (~43 kDa), representing a substantially larger fusion partner. Structural studies indicate that eRF1 inserts into the ribosomal A-site and extends its catalytic GGQ motif toward the peptidyl transferase center (PTC) during translation termination (PMID: 26245381). Therefore, fusion of a bulky catalytic domain to eRF1 may introduce steric hindrance, potentially impairing efficient ribosome engagement or perturbing the highly constrained geometry required for accurate termination. In contrast, eRF3 primarily associates with eRF1 and the ribosomal GTPase center rather than directly engaging the PTC (PMID: 24335085), which we reasoned would provide a more permissive scaffold for accommodating ADAR2dd while minimizing interference with termination fidelity.

Regarding the role of eRF3 in eukaryotic translation termination, eRF3 is an essential termination factor that promotes efficient peptide release by stimulating eRF1 activity through GTP hydrolysis (PMID: 23963452). In mammalian cells, eRF3 has two paralogs, eRF3a (GSPT1) and eRF3b (GSPT2). eRF3a is broadly expressed across multiple tissues, whereas eRF3b expression is considerably more restricted (PMID: 15987998). We therefore selected eRF3a to maximize the robustness and applicability of RAFTER across diverse mammalian cell types.

2. It has been reported that termination efficiency is different among three stop codons (i.e., UAA, UAG, UGA). Have the authors analyzed mRNAs stratified by stop codons? It is conceivable that different stop codons have distinct dwell time for eRFs and therefore affect the modification occurrence. This result is expected to complicate the data interpretation about translation efficiency.

Author Response: We thank the reviewer for highlighting this important aspect of translation termination. To investigate whether stop codon identity influences RAFTER signal, we stratified genes according to their primary stop codon (UAA, UAG, or UGA) and compared RAFTER editing levels (EPKM) with translation measurements derived from Ribo-seq and polysome-seq datasets.

As shown in Figures R3a and R3b, RAFTER exhibited a slightly stronger correlation with Ribo-seq and polysome-seq measurements among genes terminating with the UAG stop codon. Interestingly, a similar trend was also observed when comparing Ribo-seq with polysome-seq across the same stratified groups (Fig. R3c), suggesting that this phenomenon is not unique to RAFTER but may instead reflect intrinsic differences in translation dynamics associated with specific stop codons.

These results indicate that stop codon identity may contribute to variability in translation termination and ribosome occupancy. However, the overall concordance between RAFTER and independent translation measurements remains preserved across all stop codon groups, suggesting that potential differences in eRF dwell time do not substantially alter the interpretation of RAFTER-derived translation signals.

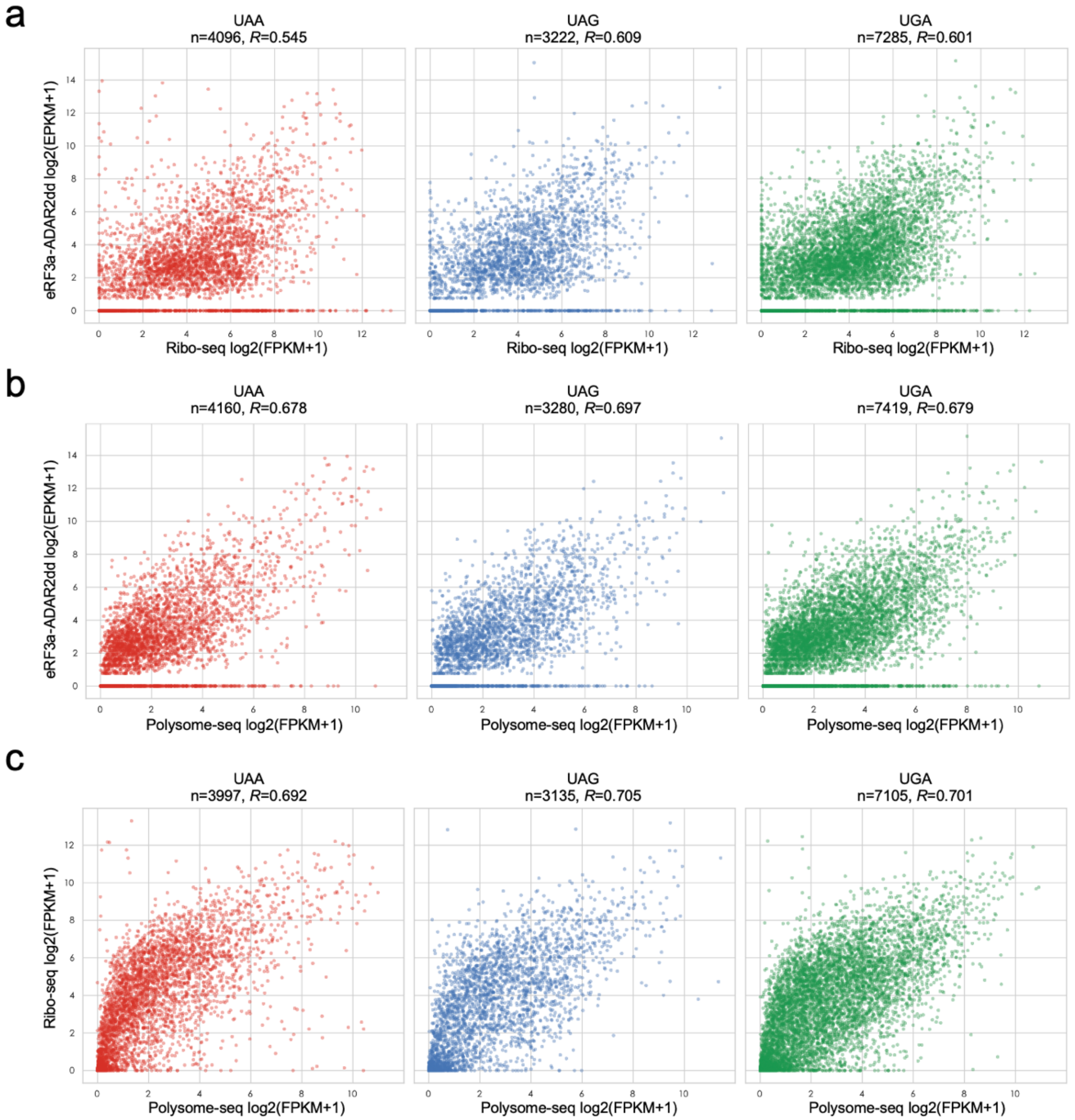

Figure R3. a-b, Scatter plots showing correlations between RNA editing levels and translation level among genes grouped by different stop codons. **a**, eRF3a-ADAR2dd vs. Ribo-seq. **b**, eRF3a-ADAR2dd vs. Polysome-seq. **c**, Scatter plots showing correlations of translation level between Ribo-seq and Polysome-seq among genes grouped by different stop codons. The number of genes (n) and Pearson correlation coefficient (R) are indicated at the top of each panel.

3. Only 1,799 annotated protein-coding genes were identified as editing targets. Why is the number so small? This is way fewer than regular Ribo-seq and raises the question of the sensitivity of RAFTER.

Author Response: We thank the reviewer for raising this important point regarding the number of identified target genes. We agree that the 1,799 detected protein-coding genes is lower than typically reported in Ribo-seq datasets and may raise questions about the sensitivity of RAFTER. We would like to clarify that the number of detected targets is strongly influenced by the expression level of the eRF3a-ADAR2dd fusion protein and reflects a trade-off between detection sensitivity and perturbation of endogenous cellular states.

To investigate this relationship, we additionally performed transient plasmid transfection to express ADAR2dd-only or eRF3a-ADAR2dd at substantially higher levels than those achieved using the TetON system (Fig. R4a). Under identical statistical thresholds (adjusted P < 0.05; editing score log2FC > 1), the number of detected protein-coding targets increased dramatically from 1,799 to 6,063 genes (Fig. R4b). These results indicate that RAFTER is capable of capturing a substantially broader translatome when the expression level of the editing enzyme is increased, suggesting that the relatively limited number of targets detected in the TetON system is likely attributable to lower expression levels rather than an intrinsic limitation in method sensitivity.

a

Samples	RNA TPM of exogenous fusion protein
Transient-ADAR2dd-only Rep.1	159618.622
Transient-ADAR2dd-only Rep.2	98186.720
Transient-eRF3a-ADAR2dd Rep.1	102693.772
Transient-eRF3a-ADAR2dd Rep.2	131008.353
TetON-ADAR2dd-only Rep.1	1711.004
TetON-ADAR2dd-only Rep.2	1701.703
TetON-eRF3a-ADAR2dd Rep.1	1661.778
TetON-eRF3a-ADAR2dd Rep.2	1594.577

b

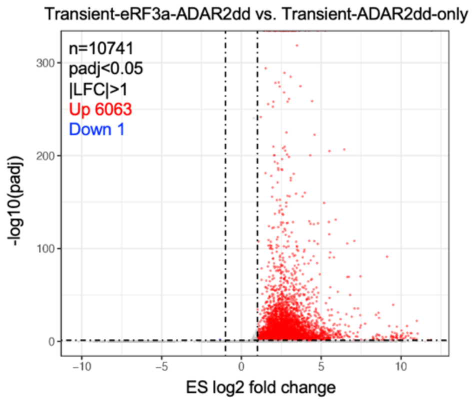

Figure R4. a, Summary of the RNA TPM of exogenous fusion protein in each sample. **b**, Volcano plots comparing the editing score (ES) of protein-coding genes between cells transiently transfected with eRF3a-ADAR2dd and ADAR2dd-only plasmids. The X and Y axes represent log2 fold change (LFC) and -log10(p-value), respectively. Statistical significance was calculated using two-sided Fisher's exact test followed by Benjamini-Hochberg correction.

However, comparative transcriptomic analyses showed that high-level transient expression of either ADAR2dd or eRF3a-ADAR2dd induced pronounced transcriptomic perturbations relative to wild-type cells. Interestingly, comparison between eRF3a-ADAR2dd and ADAR2dd revealed almost no difference (Figs. R5a-c), demonstrating the major effect was due to the overexpression of the editing enzyme instead of eRF3. In contrast, the TetON system used throughout this study caused minimal transcriptomic perturbation (Figs. R5d-f, same as Figs R1a-c), suggesting that it better preserves endogenous cellular states.

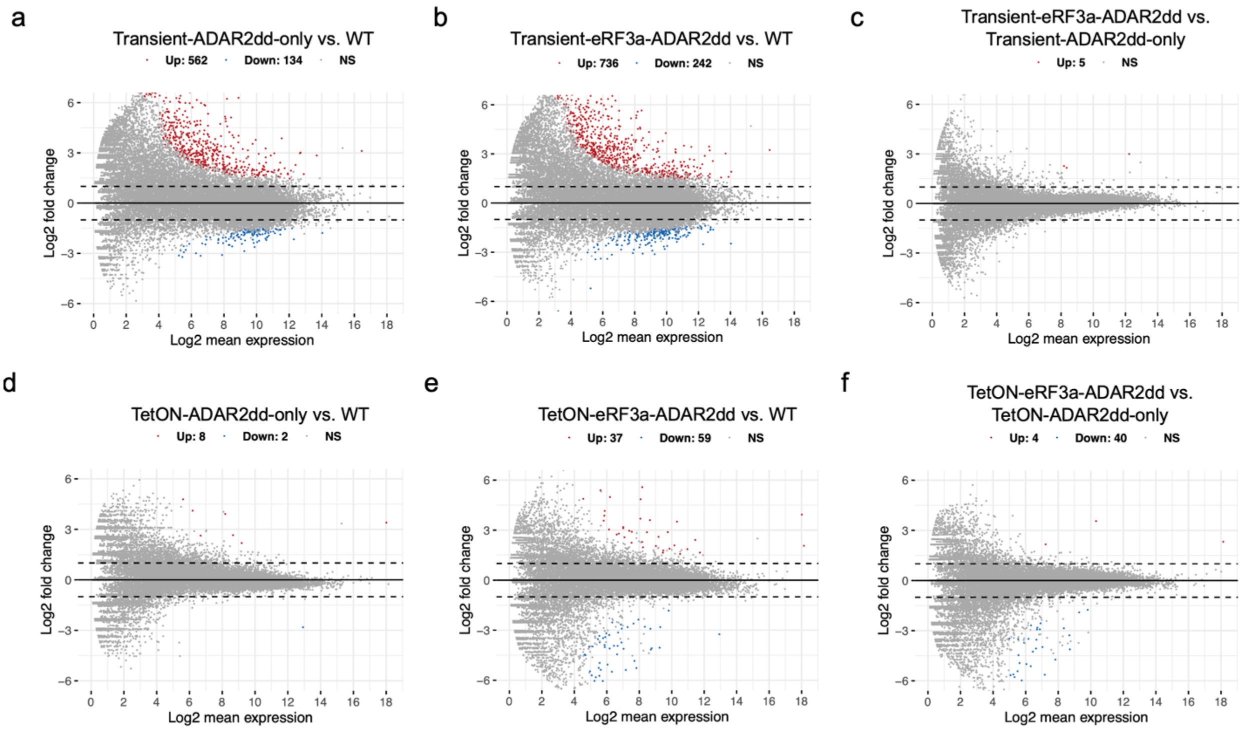

Figure R5. a-f, MA plots showing differential gene expression profiles between the indicated samples. Comparisons are specified at the top of each panel. **a**, Transient-ADAR2dd-only cells vs. wild-type (WT) cells. **b**, Transient-eRF3a-ADAR2dd cells vs. WT cells. **c**, Transient-eRF3a-ADAR2dd cells vs. Transient-ADAR2dd-only cells. **d**, TetON-ADAR2dd-only cells vs. WT cells. **e**, TetON -eRF3a-ADAR2dd cells vs. WT cells. **f**, TetON -eRF3a-ADAR2dd cells vs. TetON-ADAR2dd-only cells.

Taken together, these findings indicate that the TetON-based RAFTER system represents a compromise between maximizing detection sensitivity and minimizing experimental artifacts.

4. How could the ADAR2dd-only control also show stop codon accumulation? This raises the question of the specificity of RAFTER.

Author Response: We thank the reviewer for this observation. We would like to emphasize that RAFTER specificity is supported by the substantially stronger enrichment of editing events around stop codons observed in the eRF3a-ADAR2dd samples compared to the ADAR2dd-only control. Moreover, in eRF3a-ADAR2dd samples, editing events sharply decrease downstream into the 3' UTR, generating a distinct positional pattern that is not observed in the control condition.

To further address this concern, we generated a higher-resolution metaplot focusing on the region surrounding the stop codon (-500 to +500 nt; Fig. R6). Even with increased resolution, eRF3a-ADAR2dd retained clear enrichment of editing events around stop codons, whereas editing events in the ADAR2dd-only control appeared comparatively more uniformly distributed. These results further support that RAFTER provides specific enrichment near translation termination sites beyond the background editing pattern observed in the control.

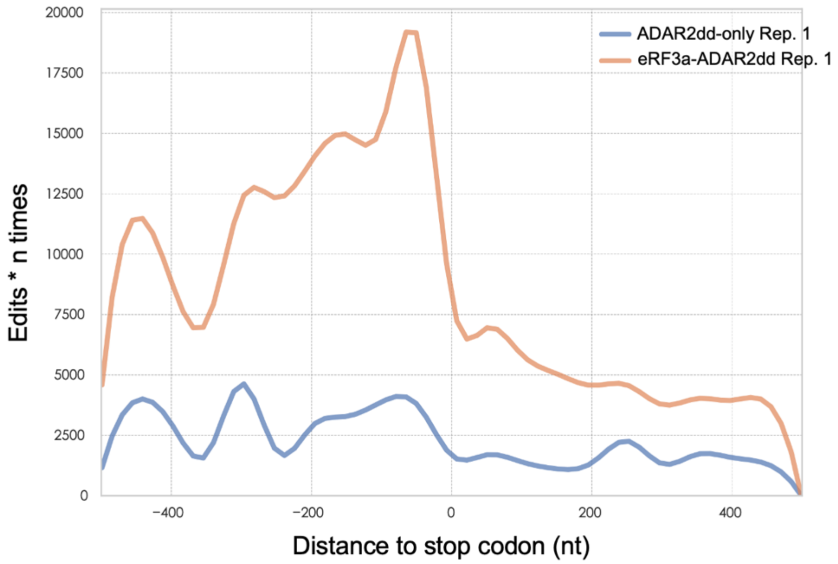

Figure R6. Metaplot showing the distribution of confident editing events (≥ 0.65 confidence score) in the flanking regions (-500 to +500 nt) of stop codons.

5. The accumulation of editing at start codons is surprising and worth in-depth analysis. Unfortunately, the authors did not pursue further if such phenomenon is true. Notably, the relative positions of edits are different between stop and start codons. While stop codons are associated with upstream peaks, start codons are accompanied with downstream edits. This cannot be explained by simple start and stop codon interaction.

Author Response: We thank the reviewer for this insightful observation regarding the editing distribution patterns around start and stop codons. Since ADAR-mediated editing does not require direct binding of the fusion protein to the edited RNA site (PMID: 32674054), we speculated that the enrichment of editing events around both start and stop codons may arise from spatial proximity between translation initiation and termination regions within mRNAs.

Regarding the reviewer's observation that stop codons appear associated with upstream editing peaks whereas start codons appear associated with downstream peaks, we speculate that this apparent asymmetry may partially reflect differences in transcript architecture rather than distinct underlying mechanisms. Specifically, CDSs (median length 1278 nt) are substantially longer than 5' UTRs (median length 203 nt) and moderately longer than 3' UTRs (median length 938 nt) in human mRNA region (PMID: 28025344). Such differences in region length may influence the distribution of detectable editing events surrounding start and stop codons and consequently affect the apparent peak positions in metagene analyses. In addition, binning and cubic spline interpolation used for metaplot generation may further exaggerate apparent positional shifts, particularly when aggregating transcripts with highly variable UTR and CDS lengths.

6. The correlation shown in Fig. 1h-k is poor and questions whether RAFTER could be truly used to evaluate translation efficiency. It is also troubling that the negative control ADAR2dd-only also showed position correlation.

Author Response: We thank the reviewer for pointing this out. We believe there are at least three lines of evidence supporting that RAFTER can be reliably used to evaluate translation level.

First, the absolute Pearson R values of eRF3a-ADAR2dd EPKM with Ribo-seq RPKM and Polysome-seq RPKM are comparable to those reported for the published Ribo-STAMP method (PMID: 33963355)

(Table R2). Notably, these values are also comparable to the correlation observed between Ribo-seq and Polysome-seq RPKM themselves, both of which are established gold standards (Table R2).

Second, the Pearson R values between the ADAR2dd-only control EPKM and Ribo-seq RPKM is substantially smaller than that reported for the Control-STAMP-high (APOBEC1-only control) in the Ribo-STAMP study (PMID: 33963355) (Table R2). This suggests that our control exhibits lower background correlation relative to the previous method.

Third, the eRF3a-ADAR2dd construct shows a much higher increase in correlation coefficient over its respective control compared to the RiboSTAMP (Table R2). Specifically, when correlating EPKM with Ribo-seq RPKM, the Pearson R value increased by 0.227 in eRF3a-ADAR2dd compared to the ADAR2dd-only control. For Polysome-seq RPKM, the increases were 0.346. In contrast, in the Ribo-STAMP method (PMID: 33963355), the Pearson R value for RPS2-STAMP-high (RPS2-APOBEC1) EPKM only increased by 0.113 when correlated with Ribo-seq RPKM, and by 0.083 for RPS3-APOBEC1 (Table R2).

Taken together, these data demonstrate that the eRF3a-ADAR2dd substantially enhances the detection of mRNA translation level that exceeds the currently available Ribo-STAMP method.

	Compared datasets	Number of genes	Pearson R
RAFTER	ADAR2dd-only vs. Ribo-seq	14,310	0.300 (Rep. 1); 0.312 (Rep. 2)
	eRF3a-ADAR2dd vs. Ribo-seq	14,310	0.533 (Rep. 1); 0.533 (Rep. 2)
	ADAR2dd-only vs. Polysome-seq	14,310	0.287 (Rep.1); 0.294 (Rep.2)
	eRF3a-ADAR2dd vs. Polysome-seq	14,310	0.637 (Rep.1); 0.635 (Rep.2)
Ribo-STAMP	Control-STAMP-high vs. Ribo-seq	20,310	0.566
	RPS2-STAMP-high vs. Ribo-seq	20,251	0.678
	RPS3-STAMP-high vs. Ribo-seq	20,296	0.648
	RPS2-STAMP-high vs. Polysome-seq	16,126	0.735
	Ribo-seq vs. Polysome-seq	14,310	0.693

Table R2. Summary of Pearson correlation coefficients (R) for respective dataset comparisons. Ribo-STAMP data were obtained from Figures 2c-e in PMID: 33963355.

7. The reporter assay shown in Fig. 2a-b is informative but not quantitative. Ideally, positive cap&tail reporters with different translation efficiency should be included. For instance, inserting secondary structures in 5'UTR, introducing non-optimal codons in CDS, etc.

Author Response: We thank the reviewer for pointing this out. We admit that our reporter assay, as currently presented, is informative but not quantitative. The reporters used in Figures 2a-b were primarily designed to provide a validation that the eRF3a-ADAR2dd recruitment can be used to indicate successful translation. In an independent study, we are using such reporter system for screening *cis*-elements of translational regulation.

8. The results obtained from oocytes are not impressive, and appear to be less informative than Ribo-lite and T&T-seq. In fact, RAFTER requires injection of exogenous eRF3a-ADAR2dd, which is impractical in many situations such as in vivo samples. This disadvantage limits its broad application, not to mention the possibility of artifacts.

Author Response: We thank the reviewer for raising these important concerns regarding the applicability and potential limitations of RAFTER in oocytes. Regarding comparison with Ribo-lite and T&T-seq, we agree that these methods provide highly informative measurements of translation in oocytes. However, we would like to emphasize that RAFTER is designed with different experimental

trade-offs and objectives. Both Ribo-lite and T&T-seq require capture of ribosome-associated RNAs from individual oocytes, involving technically demanding workflows and specialized optimization. In contrast, the current implementation of RAFTER in oocytes requires only *in vitro* transcription and microinjection of mRNA, procedures that are broadly accessible in laboratories equipped for standard oocyte manipulation. Rather than replacing existing methods such as Ribo-lite or T&T-seq, we envision RAFTER as a complementary approach that may be advantageous in contexts where direct ribosome profiling is experimentally challenging or where cumulative translation-associated signals over time are of interest.

Reviewer #3:

In this manuscript, the authors developed RAFTER, a novel tool to measure mRNA translation by fusing the release factor eRF3a with the deaminase domain of ADAR2. The authors demonstrate that RAFTER can detect translated mRNAs through A-to-I editing at termination sites and apply this method to single mouse oocytes. The concept of using release factor with deaminase to mark translated mRNAs is interesting and could be consistent with previous Ribo-lite and T&T-seq data. However, this reviewer found critical concerns about the novelty of the methodology, robustness of the technique itself, and the biological significance. This reviewer suggests that a more specific journal may be more suitable for this manuscript.

Major comments

1. First and foremost, this method is essentially the same concept as (Ribo-)STAMP and (Ribo-)TRIBE, published in earlier papers. The difference is only in the usage of eRF3a for tagging. The authors should compare their methods with those earlier methods directly side-by-side and demonstrate that eRF3a tagging outperforms others.

Author Response: We thank the reviewer for this important comment. We agree that RAFTER shares the general conceptual framework of using RNA-editing enzymes to record translation-associated events, as previously established by Ribo-STAMP and Ribo-TRIBE. However, RAFTER differs mechanistically in that it targets translation termination through eRF3a rather than associating with ribosomal proteins during elongation.

To directly address the reviewer’s suggestion, we compared RAFTER with Ribo-STAMP study (PMID: 33963355), as datasets for Ribo-TRIBE were not publicly available. In the Ribo-STAMP system, RPS2-STAMP-high (RPS2-APOBEC1) and RPS3-STAMP-high (RPS3-APOBEC1) fusion proteins were used to generate editing signatures associated with translating ribosomes, with Control-STAMP-high (APOBEC1-only) serving as the editing control.

As summarized in Table R3 (same as Table R2), although Ribo-STAMP exhibited higher absolute Pearson correlations with Ribo-seq and Polysome-seq measurements, the Control-STAMP-high showed much higher correlation with Ribo-seq relative to the ADAR2dd-only control. Thus, when considering signal relative to background, RAFTER exhibited improved specificity. Furthermore, comparison of exogenous fusion protein expression levels provided additional support for the specificity and efficiency of RAFTER (Fig. R7). Specifically, ADAR2dd-only expression was approximately 1.2-fold higher than Control-STAMP-high, yet exhibited substantially lower background correlation with Ribo-seq. These observations suggest that RAFTER has higher specificity and comparable efficiency with fusion protein expression levels similar as Ribo-STAMP.

	Compared datasets	Number of genes	Pearson R
RAFTER	ADAR2dd-only vs. Ribo-seq	14,310	0.300 (Rep. 1);

			0.312 (Rep. 2)
	eRF3a-ADAR2dd vs. Ribo-seq	14,310	0.533 (Rep. 1); 0.533 (Rep. 2)
	ADAR2dd-only vs. Polysome-seq	14,310	0.287 (Rep.1); 0.294 (Rep.2)
	eRF3a-ADAR2dd vs. Polysome-seq	14,310	0.637 (Rep.1); 0.635 (Rep.2)
Ribo-STAMP	Control-STAMP-high vs. Ribo-seq	20,310	0.566
	RPS2-STAMP-high vs. Ribo-seq	20,251	0.678
	RPS3-STAMP-high vs. Ribo-seq	20,296	0.648
	RPS2-STAMP-high vs. Polysome-seq	16,126	0.735

Table R3. Summary of Pearson correlation coefficients (R) for respective dataset comparisons. Ribo-STAMP data were obtained from Figures 2c–e in PMID: 33963355.

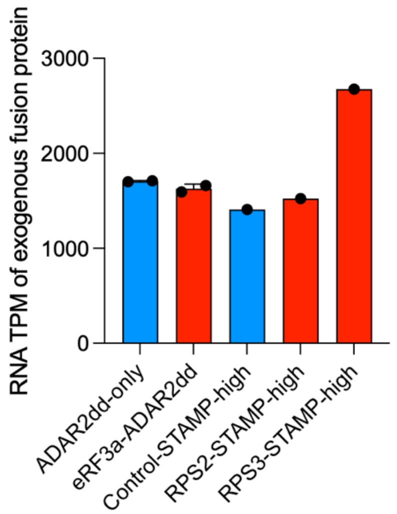

Figure R7. Barplot showing the RNA expression level (TPM) of respective exogenous fusion proteins.

2. The advantage of RAFTER over Ribo-Lite and T&T-seq is unclear from the results. Both methods have already achieved single-oocyte resolution. The authors should show the clear practical benefit of using RAFTER instead of established methods.

Author Response: We thank the reviewer for this important point. We agree that both Ribo-lite and T&T-seq have demonstrated impressive sensitivity, including single-oocyte resolution. The goal of RAFTER is not to replace these established approaches, but to provide a complementary strategy with reduced experimental complexity and broader accessibility (Table R4).

Dimension	RAFTER vs. Ribo-lite	RAFTER vs. T&T-seq
Ease of implementation	Fewer specialized steps; avoids footprint generation	Avoids separate transcriptome/translatome library preparation
Turnaround time	Shorter	Shorter
Cost	Lower reagent and labor cost	Lower reagent, labor, and sequencing cost
Technical barrier	Lower	Lower
Resolution tradeoff	Lacks codon-level resolution but retains transcriptome-wide information	Comparable gene-level resolution with simultaneous transcriptome and translation-associated information

Table R4. Comparison between RAFTER and Ribo-lite/T&T-seq.

3. Given the data shown in Fig. 1f in the metagene plot, free ADAR2dd, without tagging to eRF3a, could label the RNA around the stop codon. This suggested that 1) ADAR2dd may have the sequence

preference, that 2) this may match with the natural sequence context around the stop codon, and that 3) ultimately, off-target effects may ruin the sensitivity. This condition is not appropriate for experiments.
3a. The authors should test if such an intrinsic sequence preference and off-target effect exist in ADAR2dd.
3b. The authors should test if such a sequence preference could be minimized by the expression level control (e.g., low-level expression of the construct).
3c. If these could not be solved, the authors should avoid using eRF3a for translational profiling.
3d. ADAR2dd is the catalytic domain only, but previous reports (DOI: 10.1016/j.cell.2016.03.007) show that it still prefers dsRNA structures over ssRNA. The authors should analyze whether editing efficiency correlates with local RNA secondary structure.

Author Response: We thank the reviewer for these important comments regarding the specificity and potential off-target effects of the ADAR2dd domain. We have addressed these concerns through additional analyses and clarification as follows:

1) Intrinsic sequence preference and RNA structure dependence (Comments 3a & 3d)
We acknowledge that ADAR2dd retains preferences for specific sequence and structural contexts, particularly double-stranded RNA (dsRNA), as reported previously. However, our metagene analysis showed that editing enrichment upstream of stop codons was substantially higher in eRF3a-ADAR2dd–expressing cells than in ADAR2dd-only controls, despite slightly lower expression levels of the eRF3a-ADAR2dd fusion protein. These findings suggest that the observed editing pattern is unlikely to arise solely from the intrinsic sequence or structural preferences of ADAR2dd.

To further assess whether RNA structural features influence editing, we compared published icSHAPE scores (lower icSHAPE socre reflect nucletide with higher structures) measured in HEK293T cells (PMID: 27180905) with editing ratios detected in both ADAR2dd-only and eRF3a-ADAR2dd cells. The published icSHAPE dataset contained 4,272,570 nucleotide positions. In ADAR2dd-only cells (Replicate 1), 27,560 sites exhibited confident editing signals, of which 1,504 overlapped with icSHAPE measurements. In eRF3a-ADAR2dd cells (Replicate 1), 48,164 confident editing sites were detected, among which 4,710 overlapped with icSHAPE data. Despite the limited overlap between editing sites and available icSHAPE sites, we examined the relationship between editing ratio and icSHAPE score among overlapping sites. We observed no correlation between editing ratio and icSHAPE score in either ADAR2dd-only or eRF3a-ADAR2dd cells (Figs. R8a and R8b), suggesting that local RNA structural accessibility alone is insufficient to explain the observed editing patterns.

2) Effect of expression level on off-target editing (Comment 3b)
We acknowledge that higher expression levels of ADAR2dd may lead to increased non-specific editing. However, under conditions of higher expression achieved through transient plasmid transfection, eRF3a-ADAR2dd was able to identify substantially more protein-coding genes (Fig. R8c, same as Fig. R4b) while still exhibiting consistent stop codon-enriched editing peaks, similar to those observed in the TetON system (Fig. R8d). Nevertheless, this comes with a trade-off between increased editing efficiency and greater perturbation of cellular homeostasis, as discussed in the Author Response to Main Concern 3 raised by Reviewer 2; Therefore, we decided to use the TetON system to reduce the expression level of these constructs, which, while retaining sufficient sensitivity, largely reduced the cellular perturbation as well as the potential off-target effects.

3) Suitability of eRF3a for translational profiling (Comment 3c)
While we agree that intrinsic ADAR2dd activity contributes to background editing, our analyses indicate that the eRF3a-fused construct produces a distinct stop codon-proximal editing enrichment beyond ADAR2dd-only controls. Therefore, we believe that eRF3a remains suitable for translational profiling.

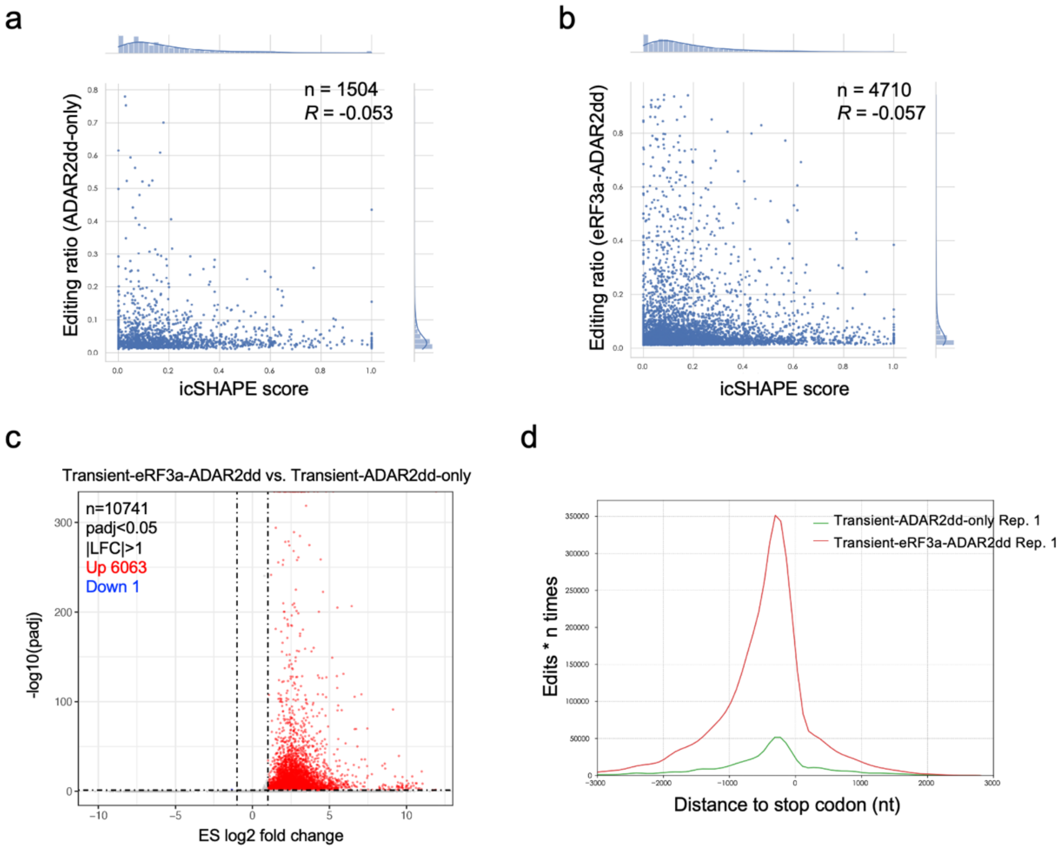

Figure R8. a-b, Scatter plots showing correlations between editing ratios and icSHAPE scores among sites overlapping between editing datasets and published icSHAPE measurements in HEK293T cells. The number of overlapping sites (n) and Pearson correlation coefficient (R) are indicated at the top right of each panel. **a**, Editing ratio in ADAR2dd-only cells vs. icSHAPE score. **b**, Editing ratio in eRF3a-ADAR2dd cells vs. icSHAPE score. **c**, Volcano plots comparing the editing score (ES) of protein coding genes between cells transiently transfected with eRF3a-ADAR2dd and ADAR2dd-only plasmids. The X and Y axes represent log2 fold change (LFC) and -log10(p-value), respectively. Statistical significance was calculated using two-sided Fisher's exact test followed by Benjamini-Hochberg correction. **d**, Metaplot showing the distribution of confident editing events (≥ 0.65 confidence score) in the flanking regions (-3000 to +3000 nt) of stop codons.

4. In Figure 1g, the authors observed A-to-I editing peaks around the start codon and discuss that this peak reflects the mRNA closed-loop structure. However, this reviewer has several concerns:
4a. This may also stem from the sequence preference, as free ADAR2dd provided the editing enrichment at the same locus.
4b. Editing also occurs at ~2000 nt from the start codon. Considering the additional RNA region in the 5′ UTR of each mRNA, this seems physically too far from the cap structure. If a closed-loop structure by cap-poly(A) interaction affects labeling in that region, labeling should occur in the cap-proximal region. However, the authors did not observe frequent labeling at the 5' UTR. The same conflict should apply to the 3′ UTR. The authors pointed out the "additional" mechanism for the closed loop. However, the evidence shown in this manuscript did not support this.
4c. The authors should use a reporter assay, as in Fig. 2a-b, to test the editing site around the start codon. The presence and absence of a cap and polyA tail in the in vitro transcribed reporter may be helpful for the discussion of the closed-loop model.
4d. As an alternative explanation to point 4a above, the weird signal at the start codon may come from the stop codons of uORFs. The authors should carefully survey this possibility.

Author Response: We thank the reviewer for these insightful comments and the suggestion to temper our claims. We have addressed these concerns as follows:

1) Sequence preference (Comment 4a): We agree that intrinsic sequence or structural preferences of ADAR2dd may contribute to baseline enrichment around start codons. However, eRF3a-ADAR2dd exhibits substantially stronger accumulation at the same region, suggesting that sequence preference alone is insufficient to explain the observed enrichment.

2) Spatial distance and the closed-loop model (Comment 4b): We acknowledge the reviewer's concern that linear distances along transcripts may appear inconsistent with a simple closed-loop interpretation. However, we would like to emphasize that the editing signal sharply decreases and approaches baseline beyond ~500 nt upstream of the start codon (Fig. R9), suggesting that the enrichment is spatially restricted rather than broadly distributed across transcript regions. In addition, our hypothesized model is based on potential spatial proximity between the start and stop codons of translating mRNAs (PMID: 32121426), rather than the canonical cap-poly(A) interaction. Given the higher-order organization of translating mRNPs, it is plausible that recruitment of eRF3a-ADAR2dd near translation termination sites could promote editing of adenosines located in physically proximal, but linearly distant, regions. Similar "in-proximity" RNA editing has previously been reported, for example in MCP-ADAR2dd systems where editing occurs on RNAs physically adjacent to MS2 loci (PMID: 32674054). We agree that our current data do not directly demonstrate such proximity and therefore revised the manuscript to present this interpretation as a hypothesis rather than a conclusion. Experimental validation of start-stop codon proximity in actively translating mRNAs remains an important direction for future investigation. We are currently collaborating with colleagues to investigate whether actively translating mRNAs can exhibit spatial proximity between start and stop codons using cryo-ET-based approaches.

3) Reporter assay (Comment 4c): As shown in our original Figure 2a-b, reporter mRNAs lacking a cap and poly(A) tail exhibited only basal editing levels at the 3' UTR. This suggests that the editing near the start codon in our reporters would likely remain at basal levels (similar to the ADAR2dd-only control) without these elements. Our model proposes a physical proximity between the start and stop codons, which may not necessarily form a canonical cap-tail closed loop.

4) uORFs (Comment 4d): Following the reviewer's suggestion, we examined whether editing signals associated with start codons could arise from translation termination at upstream open reading frames (uORFs). Among 16,923 editing events detected within 5' UTRs in eRF3a-ADAR2dd cells (Replicate 1), only 12 (~0.07%) overlapped annotated uORF regions based on the GENCODE Human Release 37 (GRCh37) annotation. These results suggest that uORF-associated translation termination is unlikely to account for the observed enrichment of editing events near start codons.

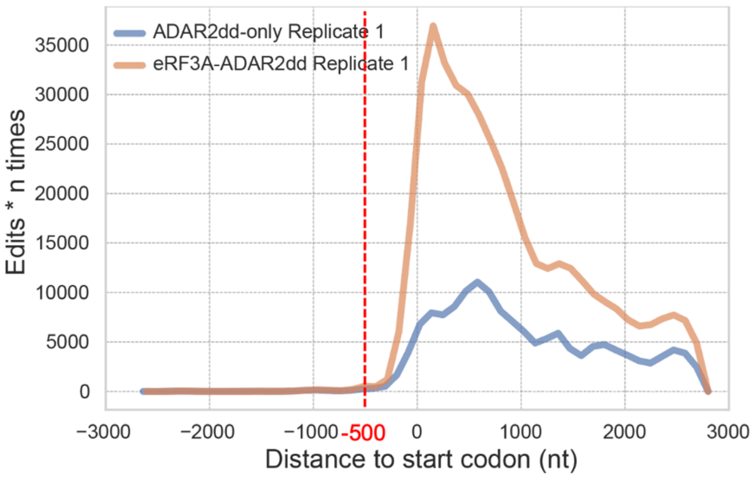

Figure R9. Metaplot showing the distribution of confident editing events (≥ 0.65 confidence score) in the flanking regions (-3000 to +3000 nt) of start codons.

5. The authors should show a coverage plot across 5'UTR-CDS-3'UTR. The current metagene plot focuses on start and stop codon regions, but editing may also occur within CDS, considering the labeling at the start codon region. This analysis would clarify whether editing is truly enriched at the stop codon or distributed throughout the CDS. If editing is also distributed within CDS, the authors should visualize metagene profiles around near-cognate stop codons and at low-optimality codons to assess the possibility that eRF3a recognizes near-cognate codons or associates with ribosomes at suboptimal codons. Such a misrecognition has been reported in earlier reports (DOI: 10.1111/febs.14709 and DOI: 10.1093/nar/gkz710). Also, the authors should test whether the CDS/UTR length, codon contents, and others may affect the editing rates.

Author Response: We thank the reviewer for these constructive suggestions.

We have included a coverage plot across the 5’ UTR-CDS-3’ UTR as requested. As shown in Figure R10a, editing events can indeed be detected within CDS regions, as anticipated by the reviewer. However, editing events substantially enriched proximal to the stop codon, particularly within the upstream region adjacent to translation termination sites in eRF3a-ADAR2dd cells, consistent with the patterns observed in our original metaplots. In contrast, such enrichment is not observed in ADAR2dd-only cells.

Regarding near-cognate stop codons and codon optimality, we agree that these are important factors that could potentially influence editing patterns through altered ribosome dynamics or termination behavior. However, RAFTER does not provide codon-level positional resolution comparable to Ribo-seq, limiting our ability to directly assess eRF3a association at individual near-cognate or low-optimality codons.

Finally, we examined whether transcript length influences editing levels. As shown in Figures R10b-d, genes with shorter CDS lengths exhibited higher editing levels in eRF3a-ADAR2dd cells (represented by EPKM), consistent with trends observed in both Ribo-seq and Polysome-seq datasets. Genes with shorter 5’ UTRs also exhibited higher editing levels, as well as higher translation levels measured by both Ribo-seq and Polysome-seq (Figs. R10e-g). Regarding 3’ UTR length, genes with shorter 3’ UTRs exhibited higher editing levels and higher translation levels measured by Polysome-seq (Figs. R10h and 10i). However, this trend was less evident in Ribo-seq data (Fig. R10j). Together, these demonstrated that the length of 5’ UTR/CDS/3’ UTR might negatively affect translation level.

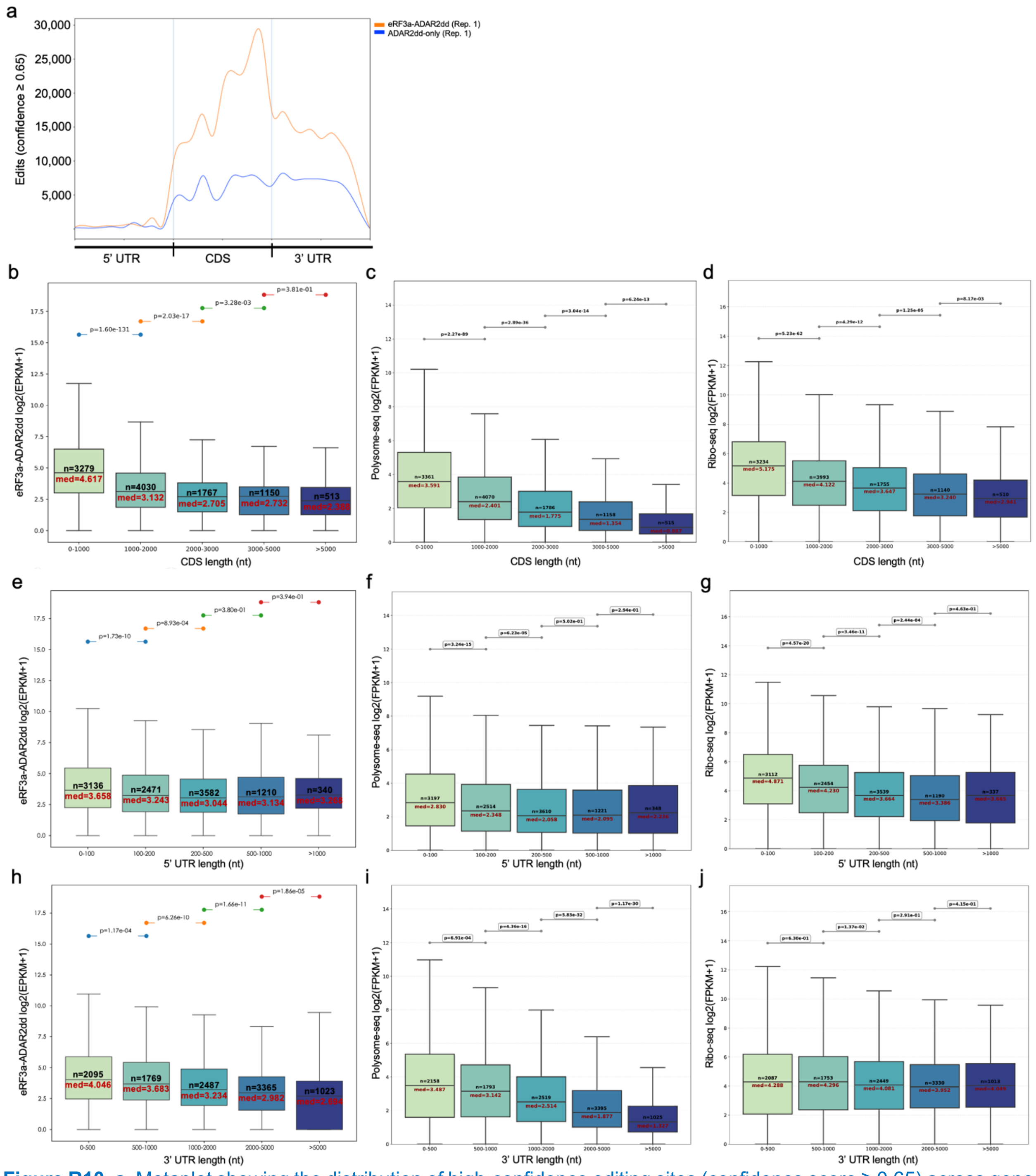

Figure R10. a, Metaplot showing the distribution of high-confidence editing sites (confidence score ≥ 0.65) across gene bodies. **b-d**, Box plots showing the distribution of editing or translation levels among genes stratified by CDS length. **b**, Editing level in eRF3a-ADAR2dd cells. **c**, Translation level measured by Polysome-seq. **d**, Translation level measured by Ribo-seq. **e-g**, Box plots showing the distribution of editing or translation levels among genes stratified by 5' UTR length. **e**, Editing level in eRF3a-ADAR2dd cells. **f**, Translation level measured by Polysome-seq. **g**, Translation level measured by Ribo-seq. **h-j**, Box plots showing the distribution of editing or translation levels among genes stratified by 3' UTR length. h, Editing level in eRF3a-ADAR2dd cells. **i**, Translation level measured by Polysome-seq. **j**, Translation level measured by Ribo-seq. The number of genes and the median editing or translation level are indicated within each figure. Statistical significance was calculated using a two-sided Wilcoxon rank-sum test.

6. In Fig. 1d, the authors claimed that ncRNA translation could be detected. The authors should show

the editing rate along the ncRNAs and short ORFs. The editing sites should correspond to the stop codon of the short ORFs in ncRNAs.

Author Response: We thank the reviewer for this suggestion. We have included representative IGV plots for five lncRNAs that exhibited significantly higher editing levels in eRF3a-ADAR2dd cells (Figs. R11a-e). However, we acknowledge that RAFTER does not provide codon-level resolution and therefore cannot directly resolve translation termination at specific stop codons within putative short ORFs encoded by these ncRNAs. Nevertheless, at the gene level, these ncRNAs exhibited higher editing levels in eRF3a-ADAR2dd cells compared with ADAR2dd-only controls, consistent with previous reports that they undergo translation.

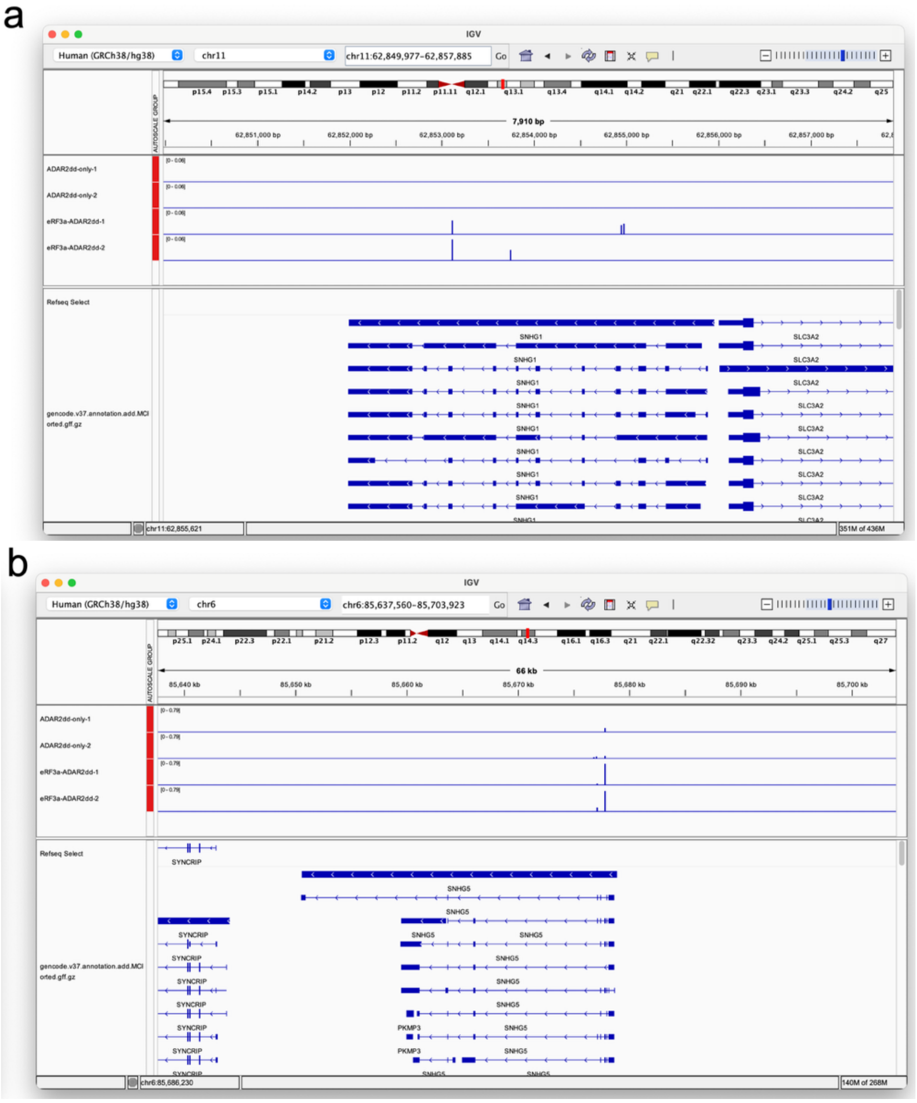

c

d

e

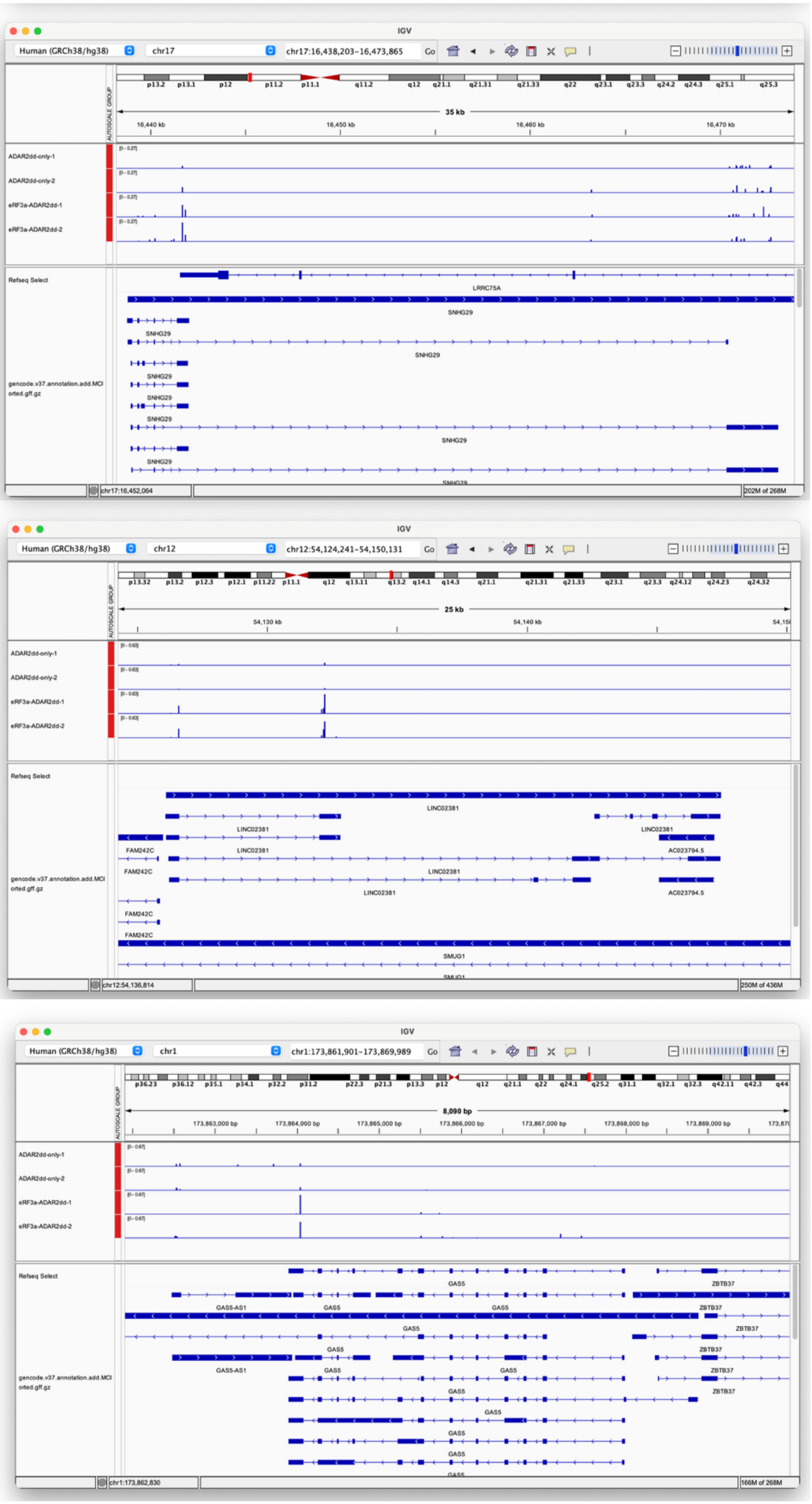

Figure R11. a-e, IGV screenshots showing identified high-confidence editing sites within 5 ncRNAs. **a**, SNHG1. **b**, SNHG5. **c**, SNHG29. **d**, LINC02381. **e**, GAS5.

7. ADAR2dd-only control shows Pearson R = 0.3 in HEK293T and R = 0.47-0.49 in oocytes (Fig. 1h, 1j, Fig. 2f, 2h). This is unexpectedly high for a negative control. The authors should discuss the reason for this high correlation. This reviewer suspects that high expression of ADAR2dd may edit exposed mRNA regions, resulting in increased off-target labeling (as suggested in the point above). Generally, this potential off-target correlation should be correlated with RNA abundance in cells. RNA abundance and Ribo-seq/polyosome-seq should show good correlation. Thus, the ADAR2dd-only may show a medium correlation (Fig. 1h, 1j, Fig. 2f, 2h). Again, the authors should revisit their conditions to minimize the off-target effects. The authors may want to investigate the mitochondria genome-encoded mRNAs, since they may serve as a good negative control.

Author Response: We thank the reviewer for these thoughtful suggestions. Indeed, in oocytes injected with ADAR2dd-only mRNA, the abundance of ADAR2dd was substantially higher than that observed in ADAR2dd-only cells induced through the TetON system in HEK293T cells (Fig. R12a). This elevated expression level could potentially contribute to increased background editing. Notably, even though eRF3a-ADAR2dd was expressed at substantially lower levels than ADAR2dd-only in injected oocytes (Fig. R12a), it still exhibited higher Pearson correlations with translation measurements. These observations suggest that RAFTER retains translation-associated specificity at lower expression levels and that background editing in oocytes may potentially be reduced by lowering the amount of injected mRNA.

Regarding mitochondrial genome-encoded transcripts, following the reviewer's suggestion, we examined genes annotated on “chrM” in the GTF files. Among the 37 mitochondrial genes, only one candidate editing event was detected (MT-ND5, position 13710), which showed no difference in editing level between cells expressing ADAR2dd-only and eRF3a-ADAR2dd (Fig. R12b).

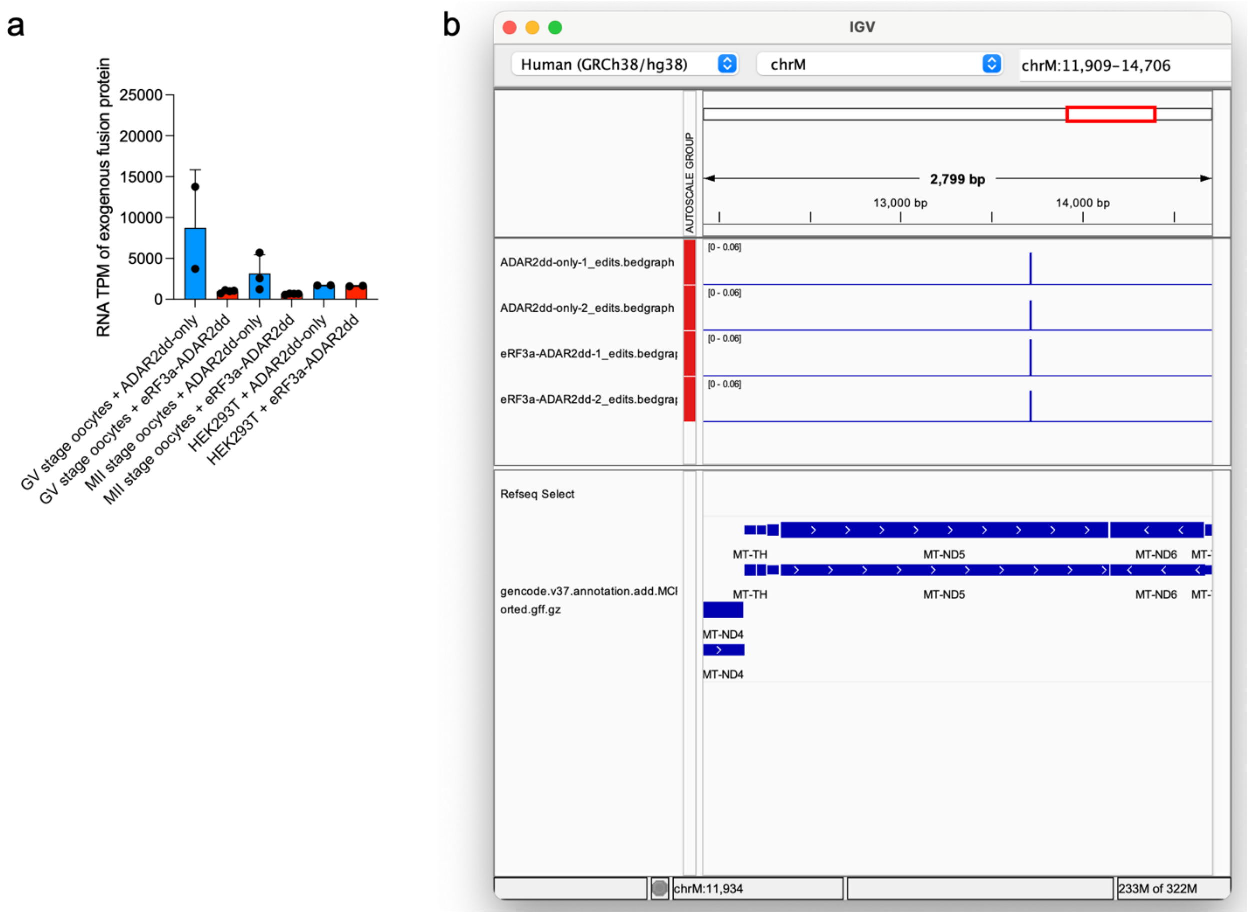

Figure R12. a, Bar plot showing the RNA abundance (TPM) of respective exogenous fusion proteins in oocytes and HEK293T cells. **b**, IGV screenshot visualizing the identified confident editing site in the MT-ND5 gene.

8. Related to the point above, this manuscript did not report translation efficiency (i.e., translation rate per mRNA). In theory, using RNA abundance and editing rate, the authors could analyze this. This should be an important aspect in the field of translation regulation.

Author Response: We thank the reviewer for this valuable suggestion. To address this point, we estimated an editing-derived translation efficiency by normalizing gene-level editing signals (EPKM) to transcript abundance (FPKM). We then compared these values with translation efficiency estimated from Ribo-seq (RPF FPKM / RNA FPKM) and Polysome-seq (polysome-associated RNA FPKM / total RNA FPKM).

As shown in Figures R13a-d, in eRF3a-ADAR2dd cells, editing-derived translation efficiency exhibited only a weak positive correlation with Ribo-seq-derived translation efficiency (R = 0.107), but a weak negative correlation with Polysome-seq-derived translation efficiency (R = -0.062). We additionally performed analogous analyses using published Ribo-STAMP datasets, as such comparisons were not reported in the original study. In contrast, RPS2-STAMP-high samples exhibited a stronger positive correlation with polysome-seq-derived translation efficiency (R = 0.211), but a slightly negative correlation with Ribo-seq-derived translation efficiency (R = -0.096).

We speculate that these differences reflect the distinct aspects of translation captured by each method. RAFTER records translation-associated events linked to termination, whereas Ribo-STAMP and polysome-seq primarily reflect the extent of polysome association, and Ribo-seq measures ribosome

occupancy along mRNAs. Therefore, agreement across these methods may not necessarily be expected.

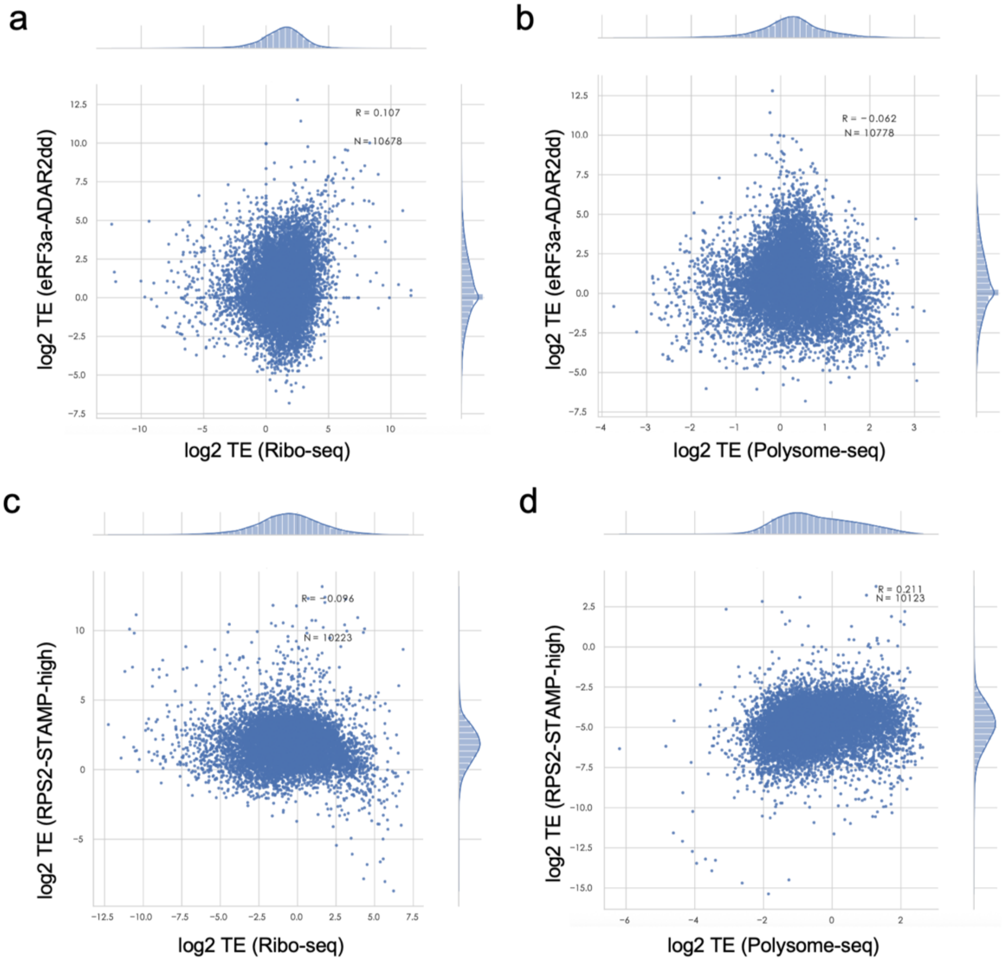

Figure R13. a-d, Scatter plots showing correlations between translation efficiency estimates derived from different methods. The number of genes (n) and Pearson correlation coefficient (R) are indicated at the top right of each panel. **a**, eRF3a-ADAR2dd vs. Ribo-seq. **b**, eRF3a-ADAR2dd vs. Polysome-seq. **c**, RPS2-STAMP-high vs. Ribo-seq. **d**, RPS2-STAMP-high vs. Polysome-seq.

9. The overexpression of termination factor (as a fusion with ADAR2d) may affect endogenous translation (or specifically termination). The authors should examine whether eRF3a-ADAR2dd fusion affects global translation level (e.g., FUNCAT, puromycin incorporation, or sucrose density gradient).

Author Response: We thank the reviewer for this important suggestion. To evaluate the effect of eRF3a-ADAR2dd overexpression on global translation, we performed polysome profiling. The resulting profiles for ADAR2dd-expressing and eRF3a-ADAR2dd-expressing cells are nearly identical (Fig. R14, same as Fig. R1d). This result suggests that eRF3a-ADAR2dd expression, at the induction level used in our study, does not impair global translation level.

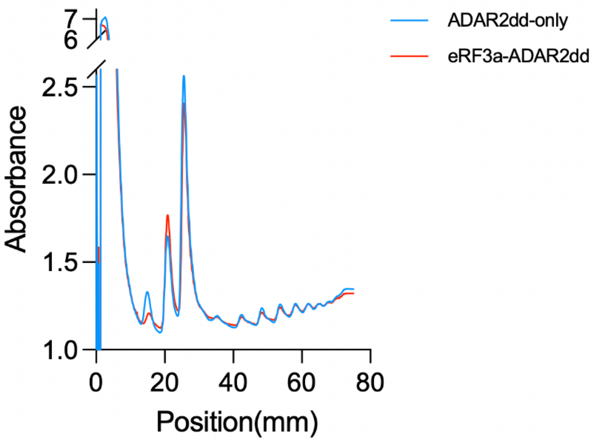

Figure R14. Polysome profiling showing the distribution of RNAs associated with free fractions (non-ribosome-bound), 40S ribosomal subunits, 60S ribosomal subunits, 80S monosomes, and polysomes in ADAR2dd-only cells (blue line) and eRF3a-ADAR2dd cells (red line).

10. In Lines 163-175, the authors summarized the published data as "increased" or "decreased". However, some of the panels did not follow this tendency. The authors should not generalize the data, ignoring the contradictions.

Author Response: We thank the reviewer for pointing this out. To address this, we revised the text to explicitly indicate which datasets support the observed trends.

Specifically, the sentence:

"Translation activity of Hmgb2 decreased at the MII stage (Supplementary Fig. 6a and 6g-i (middle panel)), whereas Mrpl10 exhibited increased translation activity at the MII stage (Supplementary Fig. 6d and 6g–i (left panel)), across all three datasets."

was revised to:

"Translation level of Hmgb2 decreased at the MII stage in RAFTER, Ribo-lite, and T&T-seq 1-oocyte datasets (Supplementary Fig. 6a and 6g-i, middle panel), whereas Mrpl10 exhibited increased translation level at the MII stage in RAFTER, Ribo-lite, and T&T-seq 10-oocyte datasets (Supplementary Fig. 6d and 6g-i, right panel)."

Minor comments

1. Figure 2a, 2b: "bp" should be "base" or "nucleotide" since it refers to single-stranded RNA.

Author Response: We thank the reviewer for pointing this out. We have replaced "bp" with "nt" throughout the revised figures where applicable, including Figures 1a and 1b and Supplementary Figures 2a and 2b.

2. Line 199-206: The source of the ADAR2dd sequence is not specified. Also, the information on the plasmid backbone was not shown. The authors should clarify these.

Author Response: We thank the reviewer for pointing these out. The nucleotide sequences of the two fusion proteins used in this study are provided in Supplementary Table 1. In addition, we have acknowledged in the revised manuscript that the plasmid backbone used in this study was obtained from Ruijun Tian's laboratory at Southern University of Science and Technology.

3. This manuscript has a lot of errors in figure citations, including:
Line 111: Fig. 1g should be Fig. 1h
Line 112: Fig. 1i should be Fig. 1j
Line 115: Fig. 1k should be removed
Line 128: Supplementary Figure 3b and 3c should be Supplementary Fig. 3c and 3d
Line 147: Supplementary Fig. 5a and 5b should be Supplementary Fig. 4a and 4b
Line 153-154: Supplementary Figure 4a and 4b should be Supplementary Figure 4c and 4d
Line 167: "the left panel" should be the "right panel"
Line 175: "the left panel" should be inserted after Supplementary Fig. 6g-i

Also, Supplementary Fig. 4e and 4f were not cited in the manuscript.

Author Response: We thank the reviewer for carefully identifying these errors. We have corrected the figure citations throughout the manuscript accordingly, including the previously omitted citations for Supplementary Figs. 4e and 4f.

4. This manuscript does not have a subheading for Introduction, Results, and Discussion. This should be included.

Author Response: We thank the reviewer for pointing this out. We have revised the manuscript accordingly.

5. Regarding the discussion in Line 47, there are a couple of reports of single-cell Ribo-Seq and several others for low-input tailored Ribo-Seq. This discussion should be reconsidered.

Author Response: We thank the reviewer for pointing this out. We have revised the statement as follows:

"Modified approaches have enabled Ribo-seq analysis in limited-input samples or even at the single-cell level, but these methods often require substantial technical optimization, which may limit their broader applicability."

6. Lines 44-45 and Lines 51-52, the same discussion is repeated. The authors should omit one.

Author Response: We thank the reviewer for pointing this out. We have omitted the redundant statement.

7. Lines 44-45 and Lines 51-52, the authors claimed that ribosome association may not always mean active translation. This reviewer agrees with this; however, the authors should cite key references to support this.

Author Response: We thank the reviewer for pointing this out. We have added two references supporting the notion that ribosome association does not necessarily indicate active translation.

8. Lines 44-45 and Lines 51-52, the ribosome on the stop codon may consist of unrecycled ribosomes (reported in many papers with Ribo-seq). Targeting the stop codon itself may not overcome this issue.

Author Response: We thank the reviewer for pointing this out. We agree with the reviewer's concern that targeting translation termination sites may not fully eliminate signals arising from unrecycled ribosomes. However, RAFTER may still provide additional information on translational regulation

beyond that obtained from conventional ribosome-dependent methods. We have added discussion of this potential limitation in the revised Discussion section.

9. Line 54, the authors should add more rationale for why targeting the termination stage may overcome the issues in the earlier methods.

Author Response: We thank the reviewer for this suggestion. We have added additional rationale in the revised manuscript: “As translation termination occurs following productive decoding of coding sequences, targeting this stage may potentially reduce signals arising from certain forms of non-productive ribosome association and provide complementary information on translational regulation.”

10. Line 120, this reviewer would say "an endogenous A site" should be "an endogenous site".

Author Response: We thank the reviewer for pointing this out. We have changed "an endogenous A site" to "an endogenous site".

11. In Fig. 1-2 and Supplementary Figs. 2, 4, 5, scatter plots may have diagonal lines; however, the detail of this was not explained in the legends. This reviewer guesses that these are regression lines, but some of the lines may not look like this (dranw far from the expectation).

Author Response: We thank the reviewer for pointing this out. The diagonal blue lines in the mentioned figures represent fitted linear regression lines, while the surrounding shaded regions indicate the corresponding 95% confidence intervals generated using the Python package Seaborn. We have clarified these details in the figure legends.

June 23, 2026

RE: Life Science Alliance Manuscript #LSA-2026-03651-TR

Prof. Wei Chen
Southern University of Science and Technology
Department of Biology
1088 Xueyuan Avenue
Shenzhen, Guangdong 518005
China

Dear Dr. Chen,

Thank you for submitting your revised manuscript entitled "RAFTER: a releasing factor tethered RNA editing system for quantifying mRNA translation". We returned this work to the original Reviewer 1. Because the original Reviewer 3 was unavailable we also sought the input of the original Reviewer 2. We sincerely appreciate the detailed assessment by Reviewer 2, and we note the clear support of Reviewer 1 for publication of this work.

In our previous letter inviting revisions based on these reviewers' initial evaluations at Molecular Systems Biology, we requested changes to comply with several of the reviewer requests. This request conveyed the minimum required by the journal, namely changes to the text, and did not preclude the addition of data to resolve these concerns. Indeed, figures included in the rebuttal document R1, R3, and R8 offer important support for the RAFTER method and directly address reviewer concerns that we felt must be resolved. In addition R4 and R5 offer valuable support that we agree would significantly strengthen this work. We concur with Reviewer 2 that the inclusion of these data is important for a reader to fully appreciate your approach to profiling translation levels. We leave the placement of these figures within the manuscript to your discretion. Overall, we would be happy to publish your paper in Life Science Alliance pending the addition of figures R1, R3, and R8, as well as formatting changes needed to comply with journal policy. The inclusion of R4 and R5 is strongly encouraged as well.

MANUSCRIPT ORGANIZATION AND FORMATTING:

To avoid unnecessary delays in the acceptance and publication of your paper, please read the following information carefully. Full guidelines are available on our Instructions for Authors page, https://www.life-science-alliance.org/authors

Submission of a paper that does not conform to Life Science Alliance guidelines will delay the acceptance of your manuscript.

-Please add a Summary Blurb/Alternate Abstract in our system.
-Please add Keywords for your manuscript in our system.
-Please add the X and Bluesky handles of your host institute/organization, as well as your own, and/or one of the authors, in our system.
-Please consult our manuscript preparation guidelines https://www.life-science-alliance.org/manuscript-prep and make sure your manuscript sections are in the correct order. Figure legends should be provided after the references and other sections before them.
-Please rename the section labeled as "Competing interests" to "Conflict of Interest."
-We encourage you to revise the figure legend for Figure S4 such that the figure panels are introduced in alphabetical order.
-Please ensure that the links included in the Data Availability section are publicly accessible.

We encourage our authors to provide original source data, particularly uncropped/-processed electrophoretic blots and spreadsheets for the main figures of the manuscript. If you would like to add source data, we would welcome one PDF/Excel-file per figure for this information. These files will be linked as supplementary "Source Data" files.

It is Life Science Alliance policy that if requested, original data images must be made available to the editors. Failure to provide original images upon request will result in unavoidable delays in publication. Please ensure that you have access to all original data images prior to final submission.

If you are planning a press release on your work, please inform us immediately to allow informing our production team and scheduling a release date.

We welcome submissions of potential cover images for the issue of LSA in which your work would appear. If you have high quality images associated with this work, please feel free to email these, with a caption, to the journal office.

LSA encourages authors to provide a 30-60 second video where the study is briefly explained. These videos will be appear embedded with the manuscript online at Life Science Alliance, and on social media to promote the published paper and authors (for examples, see https://docs.google.com/document/d/1-UWCfbE4pGcDdcgzcmiuJl2XMBJnxKYeqRvLLrLSo8s/edit?usp=sharing). Corresponding or first-authors are welcome to submit the video. Please submit only one video per manuscript. The video can be emailed to contact@life-science-alliance.org

FINAL FILES:

To upload the final version of your manuscript, please log in to your account: https://lsa.msubmit.net/cgi-bin/main.plex
You will be guided to complete the submission of your revised manuscript and to fill in all necessary information. Please get in touch in case you do not know or remember your login name.

The following items are required for acceptance.

-- An editable version of the final text (.DOC or .DOCX) is needed for copyediting (no PDFs).

-- High-resolution figure, supplementary figure and video files uploaded as individual files: See our detailed guidelines for preparing your production-ready images, https://www.life-science-alliance.org/authors

-- Summary blurb (enter in submission system): A short text summarizing in a single sentence the study (max. 200 characters including spaces). This text is used in conjunction with the titles of papers, hence should be informative and complementary to the title. It should describe the context and significance of the findings for a general readership; it should be written in the present tense and refer to the work in the third person. Author names should not be mentioned.

The license to publish form must be signed before your manuscript can be sent to production. A link to the license to publish form will be available to the corresponding author only. Please take a moment to check your funder requirements.

Reviews, decision letters, and point-by-point responses associated with peer-review at Life Science Alliance will be published, alongside the manuscript. If you do want to opt out of having the reviewer reports and your point-by-point responses displayed, please let us know immediately.

Thank you for your attention to these final processing requirements. Please revise and format the manuscript and upload materials as soon as you are able.

Thank you for this interesting contribution to the literature. We look forward to publishing your paper in Life Science Alliance.

Sincerely,

Tim Fessenden
Scientific Editor
Life Science Alliance

Reviewer #1 (Comments to the Authors (Required)):

The authors have addressed my critiques very well.

Reviewer #2 (Comments to the Authors (Required)):

This reviewer appreciates the authors' efforts to address the points raised during the previous round of review. However, this reviewer remains concerned that the major points have not been adequately addressed. Also, many of the additional analyses are described only in the response letter and have not been incorporated into the revised manuscript. This makes it difficult for readers to evaluate the validity of the authors' interpretation. For these reasons, this reviewer is unable to support publication of this manuscript.

The authors reported that the correlation coefficient for the control of Ribo-STAMP was 0.566, whereas the corresponding value reported in the original Ribo-STAMP paper was 0.32. This discrepancy may be due to differences in the Ribo-seq dataset used for the comparison. Moreover, to reduce the background signal, the authors could have used control-STAMP-low or other control samples, which are also available in GEO. This reviewer would say that the provided comparison does not allow a sufficiently fair assessment, and it remains unclear whether RAFTER indeed has a higher specificity than Ribo-STAMP.

Regarding the advantages over Ribo-Lite and T&T-Seq, this reviewer is not convinced that RAFTER has a shorter turnaround time. As the authors mentioned, the expression level of ADAR2dd-containing proteins needs to be optimized to reduce background signals and minimize effects on cell viability. Moreover, the generation of cell lines using lentiviral transduction takes substantial time.

In regions with a high editing ratio (>0.2) in Fig. R8, the icSHAPE scores appear to be lower, although no overall correlation is observed. This suggests that RNA secondary structure alone is not sufficient to explain the global editing pattern, but highly edited regions may be preferentially associated with structured RNA elements.

Regarding the editing tendency around start and stop codons, the stronger editing in eRF3a-ADAR2dd does not mean that the control has no intrinsic preference. An alternative interpretation is that eRF3a increases access to mRNAs and thereby enhances the preference of ADAR2dd-only.

This reviewer agrees that RAFTER does not provide codon-level resolution. However, the purpose of requesting analyses of near-cognate and low-optimality codons was to investigate why editing is increased within CDS regions. Similarly, for uORFs, this reviewer requested to examine whether editing is enriched around start codons associated with uORFs. Therefore, codon-level resolution is not essential for these analyses, and metagene profile analysis would still be informative.

July 7, 2026

RE: Life Science Alliance Manuscript #LSA-2026-03651-TRR

Prof. Wei Chen
Southern University of Science and Technology
Department of Biology
1088 Xueyuan Avenue
Shenzhen, Guangdong 518005
China

Dear Dr. Chen,

Thank you for submitting your Research Article entitled "RAFTER: a releasing factor tethered RNA editing system for quantifying mRNA translation". We appreciate the incorporation of new figures into the manuscript, which we hope you agree improved this manuscript considerably. It is a pleasure to let you know that your manuscript is now accepted for publication in Life Science Alliance. Congratulations on this interesting work.

Please note that both of the links to the GSA public data repository included in the Data Availability section do not appear to be functional. Currently they indicate "Wrong share code OR this preview is Cancelled". Kindly ensure these links work during the proofing processes.

Your manuscript will now progress through copyediting and proofing. The final published version of your manuscript will be deposited by us to PubMed Central upon publication.

Your article will publish open access upon publication under a CC-BY license.

Reviews, decision letters, and point-by-point responses associated with peer-review at Life Science Alliance will be published, alongside the manuscript. If you do want to opt out of having the reviewer reports and your point-by-point responses displayed, please let us know immediately.

IMPORTANT: If you will be unreachable at any time, please provide us with the email address of an alternate author. Failure to respond to routine queries may lead to unavoidable delays in publication.

Scheduling details will be available from our production department. You will receive proofs shortly before the publication date. Only essential corrections can be made at the proof stage so if there are any minor final changes you wish to make to the manuscript, please let the journal office know now.

DISTRIBUTION OF MATERIALS:
Authors are required to distribute freely any materials used in experiments published in Life Science Alliance. Authors are encouraged to deposit materials used in their studies to the appropriate repositories for distribution to researchers.

You can contact the journal office with any questions, contact@life-science-alliance.org

Again, congratulations on a very nice paper. I hope you found the review process to be constructive and are pleased with how the manuscript was handled editorially. We look forward to future exciting submissions from your lab.

Sincerely,

Tim Fessenden
Scientific Editor
Life Science Alliance
